# Formal total synthesis of macarpine via a Au(I)-catalyzed 6-*endo*-*dig* cycloisomerization strategy

**DOI:** 10.3762/bjoc.18.169

**Published:** 2022-11-23

**Authors:** Jiayue Fu, Bingbing Li, Zefang Zhou, Maosheng Cheng, Lu Yang, Yongxiang Liu

**Affiliations:** 1 Key Laboratory of Structure-Based Drug Design and Discovery (Shenyang Pharmaceutical University), Ministry of Education, Shenyang 110016, P. R. China; 2 Wuya College of Innovation, Shenyang Pharmaceutical University, Shenyang 110016, P. R. China; 3 Institute of Drug Research in Medicine Capital of China, Benxi 117000, P. R. China

**Keywords:** benzo[*c*]phenanthridine alkaloids, 1,5-enyne, formal total synthesis, gold catalysis, macarpine

## Abstract

The formal total synthesis of macarpine was accomplished by the construction of a naphthol intermediate in Ishikawa’s synthetic route with two different synthetic routes. The convergent synthetic strategies feature the utilization of Au(I)-catalyzed cycloisomerizations of a 1,5-enyne and alkynyl ketone substrates, which were prepared by Sonogashira coupling reactions.

## Introduction

**Scheme 1:**
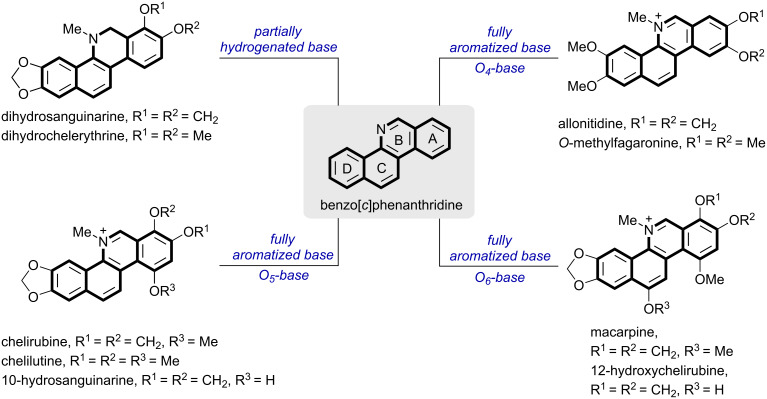
Classification of benzo[*c*]phenanthridine alkaloids.

Benzo[*c*]phenanthridine alkaloids are an ancient and influential category of isoquinoline alkaloids, mainly found in Papaveraceae and Rutaceae (Scheme 1) [1–2]. According to their oxidation states, benzo[*c*]phenanthridine alkaloids can be divided into two types: partially hydrogenated base and fully aromatized base, in which natural fully aromatic alkaloids can be further classified into three subclasses: *O*_4_-base, *O*_5_-base, and *O*_6_-base [3].

Among these alkaloids macarpine is the most oxidized tetracyclic alkaloid with many bioactivities, including anesthesia, anticancer, anti-inflammatory [4–8], insecticidal, fungicidal, etc [9]. In addition to the above-mentioned activities, macarpine was also used as a DNA probe for flow cytometry and fluorescence microscopy due to its fluorescent properties [10]. Despite some research on the activities of macarpine had been performed, a more in-depth evaluation of the biological activities was still limited due to the need of its isolation from natural sources. Inspired by the requirement of further biological evaluation, the chemical syntheses of macarpine have been developed rapidly in the last three decades.

**Scheme 2:**
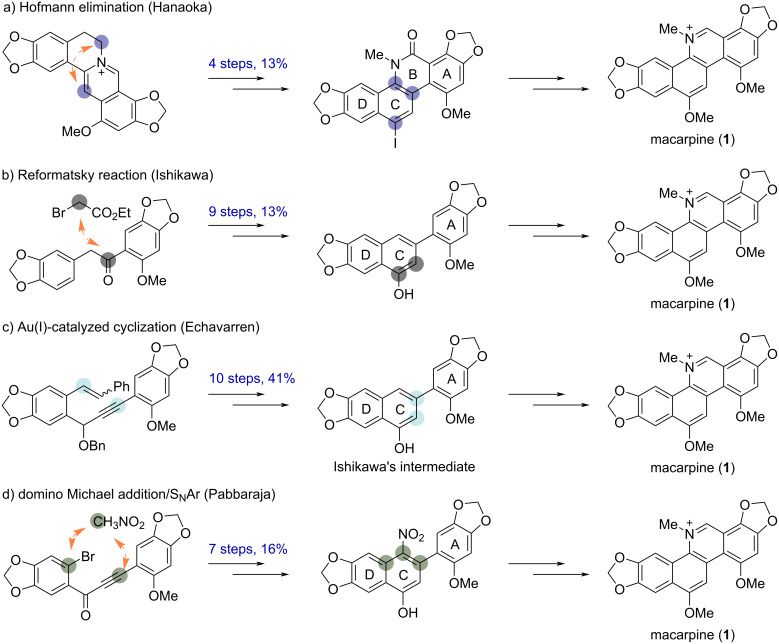
Representative synthetic strategies for macarpine (**1**).

The benzo[*c*]phenanthridine skeleton consists of a phenanthridine (rings A, B, C) and a benzene (ring D), and most of the synthetic routes were completed in the last step by constructing ring B or ring C. Some representative examples and their key strategies are summarized in Scheme 2. In 1989, Hanaoka and co-workers developed the total synthesis of macarpine by Hofmann elimination from protoberberine by introducing rings B and C (Scheme 2a) [11]. In 1995, Ishikawa and co-workers accomplished the total synthesis via a Reformatsky reaction and aromatic nitrosation through the building of rings B and C (Scheme 2b) [12]. In 2010, Echavarren and co-worker completed the formal total synthesis via a Au(I)-catalyzed cyclization (Scheme 2c) [13]. In 2018, Pabbaraja and co-workers disclosed the synthesis of macarpine by constructing ring C through the domino Michael addition/S_N_Ar reaction of nitromethane to an ynone precursor (Scheme 2d) [14].

## Results and Discussion

The efforts on developing efficient synthetic strategies to access macarpine never ceased during the last decades, and we have joined this meaningful research. Herein, a strategy involving the synthesis of an intermediate reported by Ishikawa in 1995 in the total synthesis of macarpine [12] is proposed via a Au(I)-catalyzed cycloisomerization reaction.

**Scheme 3:**
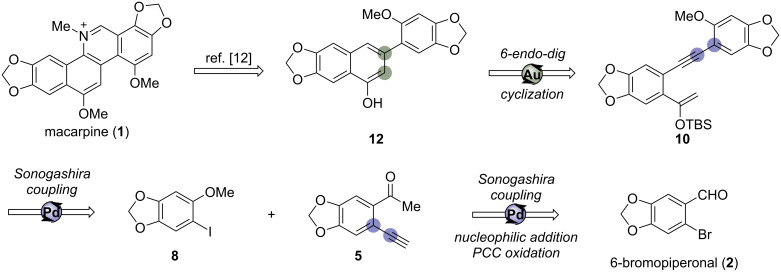
Retrosynthetic analysis of marcarpine precursor **12** for a partial synthesis.

Retrosynthetically, the target molecule macarpine (**1**) could be disconnected into naphthol **12** (Scheme 3), a key intermediate reported by Ishikawa in the total synthesis of macarpine. This intermediate could be synthesized from silyl enol ether compound **10** via the Au(I)-catalyzed cycloisomerization reaction developed by our group [15]. The compound **10** could be constructed by the Sonogashira coupling reaction from readily prepared iodoarene **8** [12,16] and ketone **5**, which could be synthesized by using cheap 6-bromopiperonal (**2**) as the starting material.

**Scheme 4:**
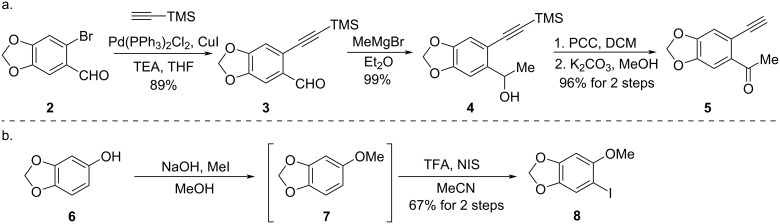
Syntheses of precursors **5** and **8**.

To attempt the proposed synthetic strategy, ketone **5** and iodoarene **8** were prepared by following the synthetic route outlined in Scheme 4. Ketone **5** was prepared in a four-step procedure. Firstly, a Sonogashira coupling between 6-bromopiperonal (**2**) and trimethylsilylacetylene was performed to furnish aldehyde **3** [17–18] in 89% yield. A following nucleophilic addition reaction of aldehyde **3** by methylmagnesium bromide (MeMgBr) gave alcohol **4** in 99% yield, which was oxidized by pyridinium chlorochromate (PCC) leading to the formation of ketone compound and the deprotection of the silyl group was accomplished in the presence of potassium carbonate (K_2_CO_3_) and methanol to provide the terminal alkyne **5** in 96% yield in two steps. The iodoarene **8** [12,16] was facilely synthesized from sesamol (**6**) via methylation and iodination in an overall yield of 67%.

**Scheme 5:**
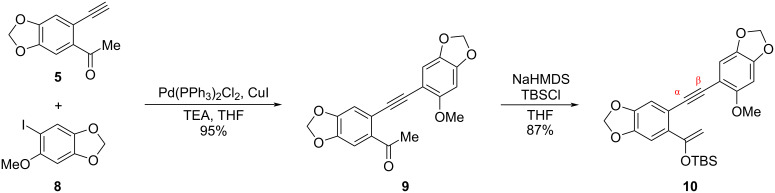
Synthesis of enol silyl ether **10**.

With the building blocks **5** and **8** in hand, ketone **9** was prepared via a palladium-catalyzed Sonogashira coupling reaction in a yield of 95%. The precursor **10** for the gold(I)-catalyzed [19–24] cycloisomerization was then synthesized by treating ketone **9** with sodium bis(trimethylsilyl)amide (NaHMDS) and *tert*-butyldimethylsilyl chloride (TBSCl) (Scheme 5).

To find the best cycloisomerization conditions, the 1,5-enyne substrate **10** was subjected to different reaction conditions as listed in Table 1. It was observed that [1,3-bis(2,6-diisopropylphenyl)imidazol-2-ylidene]gold(I) chloride (IPrAuCl) itself failed to catalyze the cycloisomerization (Table 1, entry 1). Evaluation of a number of silver salts illustrated that silver hexafluoroantimonate (AgSbF_6_) was the optimal additive to activate the gold catalyst (Table 1, entries 2, 3, and 7). Screening of the other ligands of Au(I) catalysts, including triphenylphosphane (Ph_3_P), [1,1'-biphenyl]-2-yl-di-*tert*-butylphosphane (JohnPhos) dicyclohexyl(2',4'-diisopropyl-3,6-dimethoxy-[1,1'-biphenyl]-2-yl)phosphane (BrettPhos) (Table 1, entries 4–6) revealed that 1,3-bis(2,6-diisopropylphenyl)imidazol-2-ylidene (IPr) was still the best one (Table 1, entry 7). Neither decreasing nor increasing the loading of the catalyst gave better yields (Table 1, entries 8 and 9). Examination of the reaction time showed that 2 h was the shortest reaction time and that extending the reaction time did not help to increase the yield (Table 1, entries 10 and 11). Lowering or raising the reaction temperature resulted in lower yields (Table 1, entries 12 and 13). The solvent had less effect on the reaction, and combining various factors, DCM was used for the reaction (Table 1, entries 14 and 15). When AgSbF_6_ was utilized as the sole catalyst, not any product was generated indicating cationic Au(I) was the true catalyst (Table 1, entry 16). A control experiment using 2,6-di-*tert*-butylpyridine as a proton scavenger in the IPrAuCl/AgSbF_6_ system provided the product in good yield, which excluded the influence of trace amounts of acids on the reaction (Table 1, entry 17).

**Table 1:** Optimization of the Au(I)-catalyzed cycloisomerization conditions.

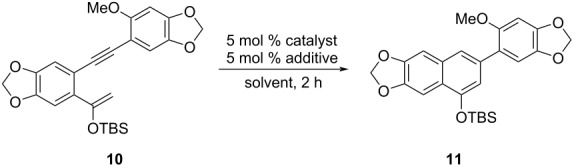					

entry	catalyst	solvent	additive	*T* (°C)	yield (%)

1	IPrAuCl	DCM	–	23	0
2	IPrAuCl	DCM	AgOTf	23	61
3	IPrAuCl	DCM	AgCO_2_CF_3_	23	23
4	Ph_3_PAuCl	DCM	AgSbF_6_	23	77
5	JohnPhosMeCNAuSbF_6_	DCM	–	23	64
6	BrettPhosAuCl	DCM	AgSbF_6_	23	45
**7**	**IPrAuCl**	**DCM**	**AgSbF** **_6_**	**23**	**82**
8	IPrAuCl	DCM	AgSbF_6_	23	68^a^
9	IPrAuCl	DCM	AgSbF_6_	23	82^b^
10	IPrAuCl	DCM	AgSbF_6_	23	57^c^
11	IPrAuCl	DCM	AgSbF_6_	23	81^d^
12	IPrAuCl	DCM	AgSbF_6_	0	63
13	IPrAuCl	DCM	AgSbF_6_	40	72
14	IPrAuCl	toluene	AgSbF_6_	23	82
15	IPrAuCl	THF	AgSbF_6_	23	80
16	–	DCM	AgSbF_6_	23	0
17	IPrAuCl	THF	AgSbF_6_	23	80^e^

^a^3 mol % IPrAuCl and 3 mol % AgSbF_6_ were used. ^b^10 mol % IPrAuCl and 10 mol % AgSbF_6_ were used. ^c^The reaction was run for 1 h. ^d^The reaction was run for 3 h. ^e^5 mol % 2,6-di-*tert*-butylpyridine was added. IPr = [1,3-bis(2,6-diisopropylphenyl)imidazol-2-ylidene]. JohnPhos = [[1,1'-biphenyl]-2-yldi-*tert*-butylphosphane]. BrettPhos = [dicyclohexyl(2',4'-diisopropyl-3,6-dimethoxy-[1,1'-biphenyl]-2-yl)phosphane].

**Scheme 6:**
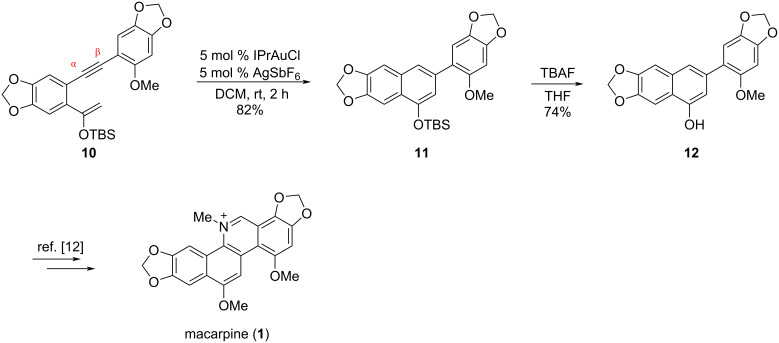
Formal total synthesis of macarpine (**1**).

The Au(I)-catalyzed cycloisomerization reaction of substrate **10** occurred under the catalysis of 5 mol % [1,3-bis(2,6-diisopropylphenyl)imidazol-2-ylidene]gold(I) chloride (IPrAuCl) and 5 mol % silver hexafluoroantimonate (AgSbF_6_) [25–26] in anhydrous DCM at room temperature for 2 h forming a benzene ring smoothly, leading to the exclusive formation of biaryl intermediate **11** in a yield of 82%. It is worth noting that the methoxy substitution in the substrate played a crucial role in controlling the selectivity of the cycloisomerization according to our previous study [15]. It was rationalized that the electron-donating phenyl ring enabled the coordination of the alkyne with the Au^+^ complex in the α-position, which promoted the silyl ether to attack the β-position of the alkyne to promote a 6-*endo*-*dig* cyclization. Next, compound **11** was subjected to a solution of tetrabutylammonium fluoride (TBAF) in tetrahydrofuran (THF), resulting in the formation of naphthol **12** [12–13], a key intermediate in the previous total synthesis of macarpine (**1**) reported by Ishikawa (Scheme 6).

To simplify the synthetic procedure, a more straightforward strategy was proposed by using alkynyl ketone **9** [27–29] as the substrate for the gold-catalyzed cycloisomerization in the presence of protonic acid. It was supposed that alkynyl ketone **9** would undergo enolization under the acidic conditions, followed by a gold-catalyzed cycloisomerization to provide the naphthol **12**.

To test the idea, alkynyl ketone **9** was subjected to different reaction conditions as listed in Table 2. It was observed that both the acids and the temperatures had a great influence on the cycloisomerization. An attempt was also made by using only *p*-toluenesulfonic acid (TsOH) in the cycloisomerization step, but no corresponding product was obtained. Finally, the optimal conditions for the Au(I)-catalyzed cycloisomerization of alkynyl ketone **9** were determined as to stir the substrate under the catalysis of 5 mol % IPrAuCl/AgSbF_6_ with 2 equiv of TsOH as the additive at 70 °C for 2 h (Table 2, entry 3). It is notable that our synthetic route to naphthol **9** is shorter and proceeds with higher yield (5 steps, 59% yield) than Ishikawa’s route (9 steps, 13% yield).

**Table 2:** Reaction condition optimization of the cycloisomerization of alkynyl ketone **9**.

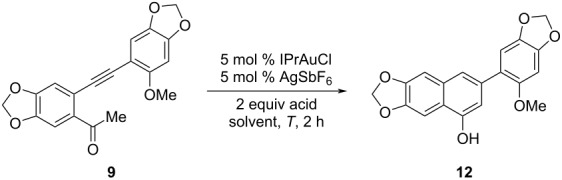				

entry	acid	solvent	*T* (°C)	yield (%)

1	TsOH	DCM	23	0
2	TsOH	DCM	40	0
**3**	**TsOH**	**DCE**	**70**	**73**
4	TFA	DCE	70	50
5	AcOH	DCE	70	24
6	PhCO_2_H	DCE	70	39

## Conclusion

In summary, the formal total synthesis of the natural product macarpine was achieved through two synthetic routes by synthesizing Ishikawa’s naphthol intermediate via Au(I)-catalyzed cycloisomerizations. Compared to the route reported in the literature, these routes are more concise and easier to perform. This gold-catalyzed strategy provides a new approach to macarpine and related benzo[*c*]phenanthridine alkaloids and the application of this strategy to access benzo[*c*]phenanthridine derivatives and further assessments of their bioactivities are currently in progress in our laboratory.

## Supporting Information

File 1 Synthetic procedures and characterization data for compounds **3**–**5**, **8**–**12**, and their ^1^H NMR and ^13^C NMR spectra.
