# Supplementary material for: Formal total synthesis of macarpine via a Au(I)-catalyzed 6-endo-dig cycloisomerization strategy
Source: Beilstein J Org Chem. 2022 Nov 23;18:1589–95. doi: 10.3762/bjoc.18.169 (PMC9704023; doi:10.3762/bjoc.18.169)
Supplement: File 1 — Synthetic procedures and characterization data for compounds 3–5, 8–12, and their 1H NMR and 13C NMR spectra. [file Beilstein_J_Org_Chem-18-1589-s001.pdf]

BEILSTEIN JOURNAL OF ORGANIC CHEMISTRY

Supporting Information

for

Formal total synthesis of macarpine via a Au(I)-catalyzed 6-*endo-dig* cycloisomerization strategy

Jiayue Fu, Bingbing Li, Zefang Zhou, Maosheng Cheng, Lu Yang and Yongxiang Liu

Beilstein J. Org. Chem. **2022,** *18,* 1589–1595. doi:10.3762/bjoc.18.169

Synthetic procedures and characterization data for compounds 3–5, 8–12, and their ^{1}H NMR and ^{13}C NMR spectra

License and Terms: This is a supporting information file under the terms of the Creative Commons Attribution License (https://creativecommons.org/licenses/by/4.0). Please note that the reuse, redistribution and reproduction in particular requires that the author(s) and source are credited and that individual graphics may be subject to special legal provisions.
The license is subject to the *Beilstein Journal of Organic Chemistry* terms and conditions: (https://www.beilstein-journals.org/bjoc/terms)

Table of contents

1. General information S2

2. Procedures for the preparation of compounds **3–5** and **8–12** and characterization data S2

3. References S10

4. NMR spectra S11

1. General information

Unless otherwise stated, the reagents were commercially available and can be used without further purification. THF and Et_2O were distilled from sodium under a nitrogen atmosphere. DCM was distilled from calcium hydride (CaH) under a nitrogen atmosphere. TLC analysis of the reaction mixtures was performed on Dynamicadsorbents silica F-254 TLC plates. Flash column chromatography was performed on Zeoprep 60 (200–300 mesh) silica gel. ^{1}H and ^{13}C NMR spectra were recorded on a Bruker Avance-III 600 spectrometer with reference to $CDCl_3$ and DMSO-d_6. HRMS–ESI were recorded on Bruker micro-TOFQ-Q instrument. IR spectra were recorded on Bruker IFS 55 spectrometer. Melting points were tested on a Thomas Hoover capillary melting point apparatus.

2. General procedures for the preparation of 3–5, 8–12 and characterization data

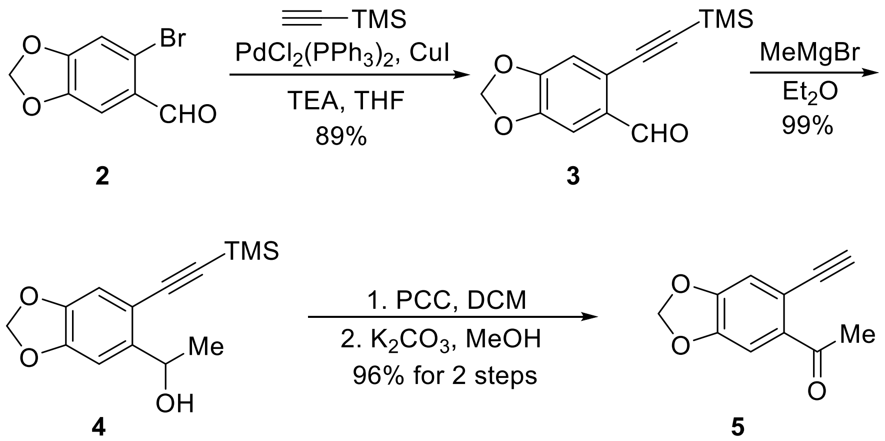

In a manner analogous to literature[s1], a magnetically stirred emulsion of 6-bromopiperonal (15.00 g, 65.79 mmol), $Pd(Ph_3P)_2Cl_2$ (1.39 g, 1.97 mmol), and CuI (1.25 g, 6.58 mmol) in degassed THF (200.0 mL) at 60 °C under an atmosphere of nitrogen was treated with TEA (18.5 mL, 131.58 mmol) and trimethylethynylsilicon (9.69 g, 98.69 mmol). The resulting mixture was stirred until the terminal alkyne was completely consumed. After the reaction is completed, the reaction solution is concentrated and purified by a flash column chromatography on silica gel to afford the product **3** (14.40 g, 58.55 mmol, 89%) as a yellow solid.

6-((Trimethylsilyl)ethynyl)benzo[*d*][1,3]dioxole-5-carbaldehyde (3)

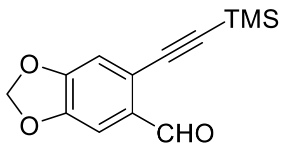

3

Yellow solid (14.40 g, 58.55 mmol, 89%) (EtOAc/petroleum ether = 1:50); Mp 113.2 – 114.7 °C; ^{1}H NMR (600 MHz, DMSO-d_6) δ 10.23 (s, 1H), 7.23 (s, 1H), 7.16 (s, 1H), 6.20 (s, 2H), 0.29 – 0.20 (m, 9H); ^{13}C NMR (150 MHz, DMSO-d_6) δ 189.1, 152.4, 148.9, 132.0, 122.0, 112.0, 105.3, 103.0, 100.6, 100.2, -0.3; IR (thin film, cm^{-1}): 3118, 2954, 2899, 2148, 1682, 1533, 1495, 1358, 1059, 996, 880, 759, 650; HRMS (ESI): *m/z* Calcd. for $C_{13}H_{15}O_3Si$ [M+H]$^+$ 247.0785, Found 247.0782. Data are in agreement with those reported in literature.[s1]

In a manner analogous to literature [s2], to a solution of compound **3** (1.92 g, 7.80 mmol) in dry Et_2O (35.0 mL) was added dropwise methylmagnesium bromide (3.0 M in Et_2O, 2.6 mL) at −78 °C with stirring under a nitrogen atmosphere. After the addition was completed, the reaction solution was allowed to warm to room temperature, and stirring was continued for 3 h. After the completion of the reaction, it was quenched with a saturated aqueous solution of NH_4Cl (20.0 mL), extracted with EtOAc (10.0 mL × 3), and washed with water. The organic layer was washed with brine and dried over anhydrous Na_2SO_4. Then, the organic layers were filtered, concentrated under reduced pressure, and purified by a flash column chromatography on silica gel to afford the product **4** (2.02 g, 7.70 mmol, 99%) as a yellow oily liquid.

1-(6-((Trimethylsilyl)ethynyl)benzo[*d*][1,3]dioxol-5-yl)ethan-1-ol (4)

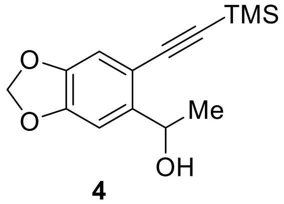

4

Yellow oily liquid (2.02 g, 7.70 mmol, 99%) (EtOAc/petroleum ether = 1:10); ^{1}H NMR (600 MHz, DMSO-d_6) δ 7.04 (s, 1H), 6.88 (s, 1H), 6.07–5.99 (m, 2H), 5.23 (d, J = 4.2 Hz, 1H), 5.03 (dd, J = 6.3, 4.3 Hz, 1H), 1.26 (d, J = 6.4 Hz, 3H), 0.26 – 0.17 (m, 9H); ^{13}C NMR (150 MHz, DMSO-d_6) δ 148.0, 145.6, 145.1, 111.0, 110.1, 104. 9, 102.9, 101.0, 96.8, 65.6, 24.4, -0.4; IR (thin film, cm^{-1}): 2944, 2832, 1663, 1483, 1449, 1371, 1252, 1114, 1020, 857, 846; HRMS (ESI): *m/z* Calcd. for $C_{14}H_{19}O_3Si$ [M+H]$^+$ 263.1098, Found 263.1090.

In a manner analogous to literature [s3], to a solution of PCC (952 mg, 4.10 mmol) in dry DCM (30.0 mL) was added compound **4** (707 mg, 2.70 mmol) in DCM at 0 °C with

stirring under a nitrogen atmosphere. After the addition was completed, the reaction solution was allowed to warm to room temperature and stirred overnight. After the reaction was completed, the reaction solution was passed through a sand funnel to obtain crude product and evaporated to dryness.

To a solution of the crude product obtained above in MeOH (40.0 mL) was added K_2CO_3 (370 mg, 2.70 mmol) and the resulting mixture was stirred at room temperature for 3 h. After completion of the reaction, it was quenched with saturated aqueous solution of NH_4Cl (5.0 mL), extracted with DCM (5.0 mL × 3), and washed with water. The organic layers were washed with brine and dried over anhydrous Na_2SO_4. Then, the organic layers were filtered, concentrated under reduced pressure, and purified by flash column chromatography on silica gel to afford the product **5** (487 mg, 2.60 mmol, 96% for 2 steps) as a yellow oily liquid.

1-(6-Ethynylbenzo[*d*][1,3]dioxol-5-yl)ethan-1-one (5)

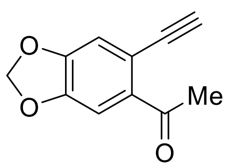

5

Yellow oily liquid (487 mg, 2.60 mmol, 96% for 2 steps) (EtOAc/petroleum ether = 1:15); ^{1}H NMR (600 MHz, DMSO-d_6) δ 7.32 (s, 1H), 7.10 (s, 1H), 6.15 (s, 2H), 4.34 (s, 1H), 2.58 (s, 3H); ^{13}C NMR (150 MHz, DMSO-d_6) δ 198.0, 150.2, 148.5, 136.6, 115.3, 113.7, 109.0, 103.1, 85.1, 82.9, 30.1; IR (thin film, cm^{-1}): 2937, 2852, 1674, 1582, 1457, 1121, 912, 891, 727; HRMS (ESI): *m/z* Calcd. for $C_{11}H_9O_3$ [M+H]$^+$ 189.0546, Found 189.0540.

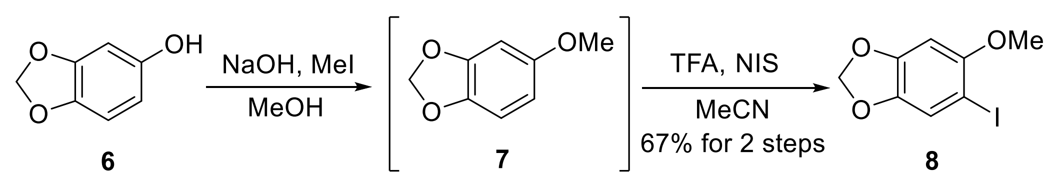

6 **7** **8**

In a manner analogous to literature [s4,s5], to a solution of sesamol (1.00 g, 7.24 mmol) and K_2CO_3 (1.00 g, 7.24 mmol) in dry MeOH (30.0 mL) was added MeI (2.5 mL, 40.10 mmol) at room temperature with stirring under a nitrogen atmosphere. After the addition was completed, the reaction solution was stirred 65 °C overnight. After the reaction was completed, the reaction solution was removed and the resulting residue was diluted with water and extracted with EtOAc (10.0 mL × 3). The combined organic

layers were dried over anhydrous Na_2SO_4 and concentrated.

To a solution of the crude product obtained above in MeCN (20.0 mL) was added NIS (2.00 g, 8.89 mmol) and TFA (0.1 mL, 1.10 mmol). The resulting mixture was stirred at 40 °C for 3 h. After completion of the reaction, it was quenched with saturated aqueous solution of $Na_2S_2O_3$ (20.0 mL), extracted with EtOAc (10.0 mL × 3), and washed with water. The organic layers were washed with brine and dried over anhydrous Na_2SO_4. Then, the organic layers were filtered, concentrated under reduced pressure, and purified by a flash column chromatography on silica gel to afford the product **8** (1.35 g, 4.85 mmol, 67% for 2 steps) as a white solid.

5-Iodo-6-methoxybenzo[*d*][1,3]dioxole (8)

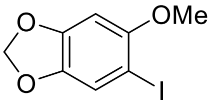

8

White solid (1.35 g, 4.85 mmol, 67% for 2 steps) (EtOAc/petroleum ether = 1:40); ^{1}H NMR (600 MHz, DMSO-d_6) δ 7.26 (s, 1H), 6.84 (s, 1H), 6.01 (s, 2H), 3.75 (s, 3H); ^{13}C NMR (150 MHz, DMSO-d_6) δ 153.7, 149.2, 142.5, 117.5, 102.2, 96.0, 73.4, 57.6. Data are in agreement with those reported in literature.[s4,s5]

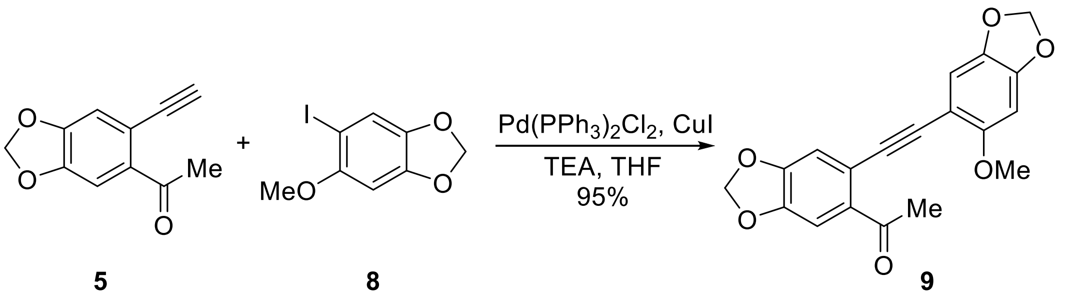

5 **8** **9**

In a manner analogous to the literature [s3], a magnetically stirred emulsion of **8** (2.00 g, 11.00 mmol), $Pd(Ph_3P)_2Cl_2$ (39 mg, 0.55 mmol), and CuI (21 mg, 1.10 mmol) in degassed THF (50.0 mL) at room temperature under an atmosphere of nitrogen was treated with TEA (7.7 mL, 55.00 mmol) and **5** (3.10 g, 11.00 mmol). The resulting mixture was stirred for 2 h sequentially. After the consumption of the starting material, the emulsion was filtered through Celite. The filtrate was concentrated under reduced pressure to obtain product **9** (3.50 g, 10.50 mmol, 95%) as a black solid.

1-(6-((6-Methoxybenzo[*d*][1,3]dioxol-5-yl)ethynyl)benzo[*d*][1,3]dioxol-5-yl)ethan-1-one (9)

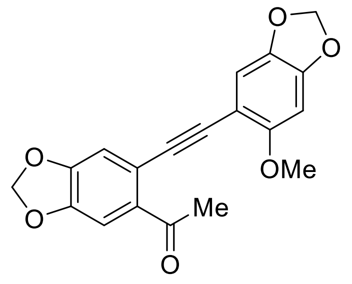

9

Black solid (3.50 g, 10.50 mmol, 95%) (EtOAc/petroleum ether = 1:20); Mp 80.0 – 80.9 °C; ^{1}H NMR (600 MHz, DMSO-d_6) δ 7.28 (s, 1H), 7.10 (s, 1H), 7.01 (s, 1H), 6.88 (s, 1H), 6.16 (s, 2H), 6.04 (s, 2H), 3.79 (s, 3H), 2.71 (s, 3H); ^{13}C NMR (150 MHz, DMSO-d_6) δ 198.3, 157.3, 150.5, 149.7, 148.2, 141.1, 135.4, 117.3, 112.6, 111.5, 108.7, 103.0, 102.2, 95.8, 91.9, 91.6, 57.0, 30.5; IR (thin film, cm^{-1}): 2944, 2833, 1472, 1418, 1448, 1268, 1195, 1113, 1020; HRMS (ESI): *m/z* Calcd. for $C_{19}H_{15}O_6$ [M+H]$^+$ 339.0863, Found 339.0859.

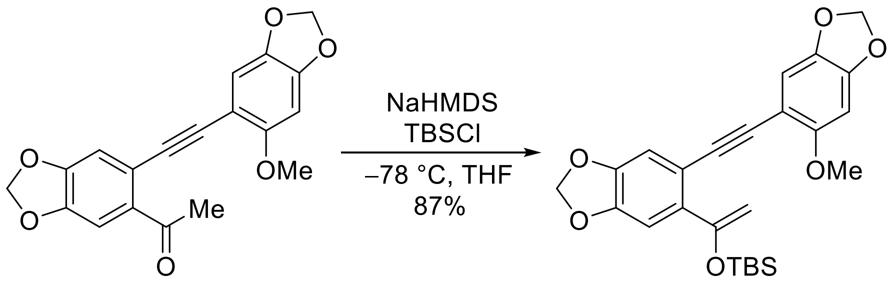

9 **10**

In a manner analogous to literature[s3], to a magnetically stirred solution of substrate **9** (1.00 g, 2.90 mmol) in degassed THF (30.0 mL) maintained at −78 ℃ under an atmosphere of nitrogen was added a solution of NaHMDS (4.4 mL, 4.40 mmol, 1.0 M) in THF dropwise and then the mixture was stirred at room temperature for 1 h. To the reaction mixture was added a solution of the TBSCl (660 mg, 4.40 mmol) in THF (10.0 mL) at −78 ℃ and the reaction mixture was stirred for 2 h at room temperature. The reaction was quenched with a saturated aqueous solution of NH_4Cl (30.0 mL) and extracted with EtOAc (10.0 mL × 3). The combined organic layers were washed with brine (20.0 mL) and dried over anhydrous Na_2SO_4, filtered, and concentrated in vacuo. The residue was purified by flash column chromatography on silica gel to afford the product **10** (1.10 g, 2.50 mmol, 87%) as a yellow solid.

***tert*-Butyl((1-(6-((6-methoxybenzo[*d*][1,3]dioxol-5-yl)ethynyl)benzo[*d*][1,3]dioxol-5-yl)vinyl)oxy)dimethylsilane (10)**

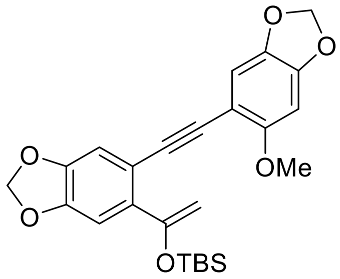

10

Yellow solid (1.10 g, 2.50 mmol, 87%) (EtOAc/petroleum ether = 1:50); Mp 90.1 – 91.5 °C; ^{1}H NMR (600 MHz, DMSO-d_6) δ 7.00 (s, 1H), 6.98 (s, 1H), 6.89 (s, 1H), 6.85 (s, 1H), 6.09 (s, 2H), 6.03 (s, 2H), 5.19 (d, J = 1.5 Hz, 1H), 4.69 (d, J = 1.5 Hz, 1H), 3.78 (s, 3H), 0.90 (s, 9H), 0.13 (s, 6H); ^{13}C NMR (150 MHz, $CDCl_3$) δ 156.8, 153.8, 148.8, 147.6, 146.8, 141.1, 135.5, 114.6, 112.4, 111.9, 107.9, 101.7, 101.6, 96.7, 95.0, 92.4, 88.8, 56.9, 25.9, -4.5; IR (thin film, cm^{-1}): 2954, 2927, 2897, 2856, 2201, 1619, 1485, 1434, 1360, 1378, 1253, 1228, 1192, 1175, 1116, 1074, 1038, 937, 860, 831, 781, 760, 686; HRMS (ESI): *m/z* Calcd. for $C_{25}H_{29}O_6Si$ [M+H]$^{+}$ 453.1728, Found 453.1718.

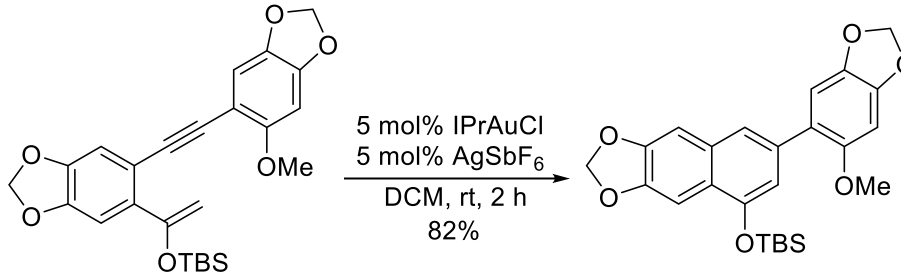

10 **11**

In a manner analogous to literature[s3], a magnetically stirred solution of substrate **10** (452 mg, 1.00 mmol) in dry DCM (10.0 mL) at room temperature under an atmosphere of nitrogen was treated with the gold catalyst, which was generated by stirring the mixture of IPrAuCl (30 mg, 0.05 mmol) and $AgSbF_6$ (17 mg, 0.05 mmol) in dry DCM (5.0 mL) at room temperature for 30 min. The resulting mixture was stirred at room temperature for 2 h and concentrated under reduced pressure. The residue was purified by flash column chromatography on silica gel to afford the product **11** (371 mg, 0.82 mmol, 82%) as a yellow oily liquid.

***tert*-Butyl((7-(6-methoxybenzo[*d*][1,3]dioxol-5-yl)naphtho[2,3-*d*][1,3]dioxol-5-yl)oxy)dimethylsilane (11)**

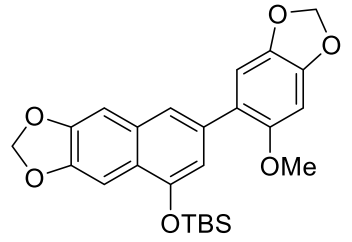

11

Yellow oily liquid (371 mg, 0.82 mmol, 82%) (EtOAc/petroleum ether = 1:10); ^{1}H NMR (600 MHz, DMSO-d_6) δ 7.37 (d, J = 1.2 Hz, 1H), 7.28 (s, 2H), 6.95 (d, J = 1.5 Hz, 1H), 6.94 (s, 1H), 6.90 (s, 1H), 6.12 (s, 2H), 6.02 (s, 2H), 3.71 (s, 3H), 1.04 (s, 9H), 0.29 – 0.23 (m, 6H); ^{13}C NMR (150 MHz, DMSO-d_6) δ 152.0, 149.8, 148.1, 147.8, 147.5, 141.6, 135.1, 132.0, 122.7, 122.4, 120.7, 114.5, 110.1, 104.4, 101.7, 98.3, 96.4, 57.0, 26.2, 18.6, -3.9; IR (thin film, cm^{-1}): 3032, 2990, 2877, 2865, 1460, 1431, 1352, 940, 757; HRMS (ESI): *m/z* Calcd. for $C_{25}H_{29}O_6Si$ [M+H]$^+$ 453.1728, Found 453.1725.

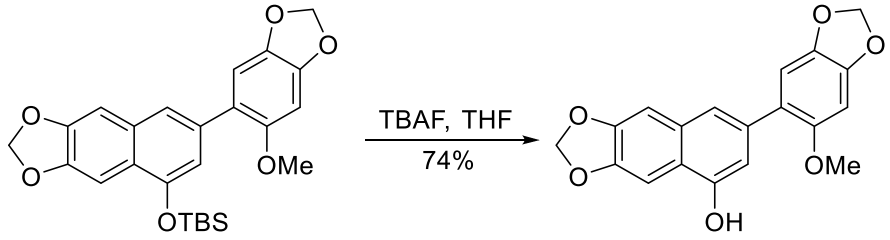

11 **12**

In a manner analogous to literature[s6], to a magnetically stirred solution of substrate **11** (362 mg, 0.80 mmol) in dry THF (5.0 mL) maintained at 0 °C under an atmosphere of nitrogen was added dropwise of a solution of TBAF (1.2 mL, 1.20 mmol, 1.0 M) in THF and then the mixture was stirred at room temperature for 30 min. The reaction was quenched with a saturated aqueous solution of NH_4Cl (10.0 mL) and extracted with EtOAc (20.0 mL × 3). The combined organic layers were washed with brine (20.0 mL) and dried over anhydrous Na_2SO_4, filtered, and concentrated in vacuo. The residue was purified by flash column chromatography on silica gel to afford the product **12** (200 mg, 0.59 mmol, 74%) as a yellow oily liquid.

7-(6-Methoxybenzo[*d*][1,3]dioxol-5-yl)naphtho[2,3-*d*][1,3]dioxol-5-ol (12)

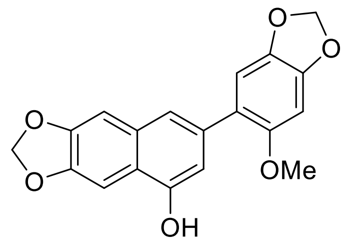

12

Yellow oily liquid (200 mg, 0.59 mmol, 74%) (EtOAc/petroleum ether = 1:15); ^{1}H NMR (600 MHz, DMSO-d_6) δ 9.85 (s, 1H), 7.35 (s, 1H), 7.21 (s, 1H), 7.18 (s, 1H), 6.89 (s, 1H), 6.87 (s, 1H), 6.86 (d, J = 1.5 Hz, 1H), 6.09 (s, 2H), 6.01 (s, 2H), 3.69 (s, 3H); ^{13}C NMR (150 MHz, DMSO-d_6) δ 152.2, 152.0, 147.9, 147.5, 146.8, 141.4, 135.3, 131.8, 123.1, 119.9, 118.6, 110.2, 109.9, 104.2, 101.6, 101.4, 98.5, 96.4, 57.1; IR (thin film, cm^{-1}): 2957, 1715, 1620, 1502, 1464, 1442, 1261, 1278, 1231, 1166, 1118, 1075, 998, 936, 849, 826., 759, 675, 646, 503; HRMS (ESI): *m/z* Calcd. for $C_{19}H_{15}O_6$ $[M+H]^+$ 339.0863, Found 339.0860. Data are in agreement with those reported in literature.[s5,s7]

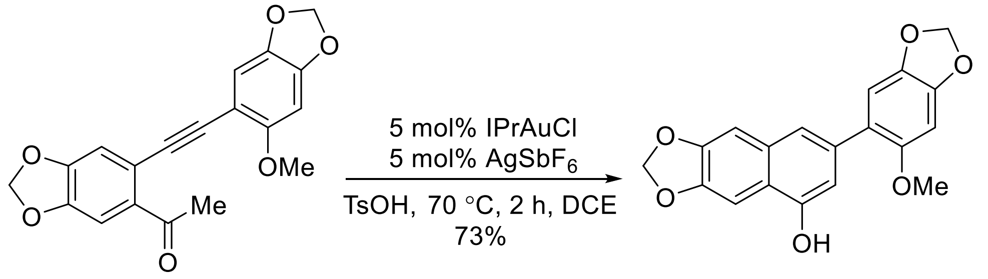

9 **12**

To a magnetically stirred solution of ketone **9** (338 mg, 1.00 mmol) in dry DCE (2 mL) at room temperature under an atmosphere of nitrogen, was added TsOH (344 mg, 2.00 mmol) dissolved in dry DCE (1.0 mL) and the mixture was stirred for 5 min. Then, the gold catalyst, which was generated by stirring the mixture of IPrAuCl (30 mg, 0.05 mmol) and $AgSbF_6$ (17 mg, 0.05 mmol) in dry DCE (2.0 mL) was added dropwise to the above solution. The resulting mixture was stirred at 70 °C for 2 h and concentrated under reduced pressure. The residue was extracted with DCM (5.0 mL × 3) and the combined organic layers were dried over anhydrous Na_2SO_4. After evaporation of the solvent under reduced pressure, the crude product was subjected to column chromatography on silica gel to give the product **12** (73%, 247 mg).

3. References

[s1]. Hashmi, A. S. K.; Hofmann, J.; Shi, S.; Schütz, A.; Rudolph, M.; Lothschütz, C.; Wieteck, M.; Buehrle, M.; Woelfle, M.; Rominger, F., *Chem. -Eur. J.*, **2013**, *19*, 382–389.

[s2]. Lu, J.; Xu, R.;Zeng, H.; Zhong, G; Wang, M; Ni, Z.; Zeng, X. *Org. Lett.*, **2021**, *23*, 3426–3431.

[s3]. Fu, J.; Li, B.; Wang, X.; Liang, Q.; Peng, X.; Yang, L.; Wan, T.; Wang, X., Lin, B.; Cheng, M.; Liu, Y. *Chin. J. Chem.*, **2022**, *40*, 46–52.

[s4]. Huang, Q.; Wang, P.; Tian, Y.; Song, N.; Ren, S.; Tao, J.; Hang, K.; Li, M. *Synlett*, **2015**, *26*, 1385–1390.

[s5]. Ishikawa, T.; Saito, T.; Ishii, H. *Tetrahedron*, **1995**, *51*, 8447–8458.

[s6]. Tanoguchi, M.; Arimoto, M.; Saika, H.; Yamaguchi, H. *Chem. Pharm. Bull.*, **1987**, *35*, 4162–4168.

[s7]. Solorio-Alvarado, C. R.; Echavarren, A. M. *J. Am. Chem. Soc.*, **2010**, *132*, 11881–11883.

4. NMR spectra

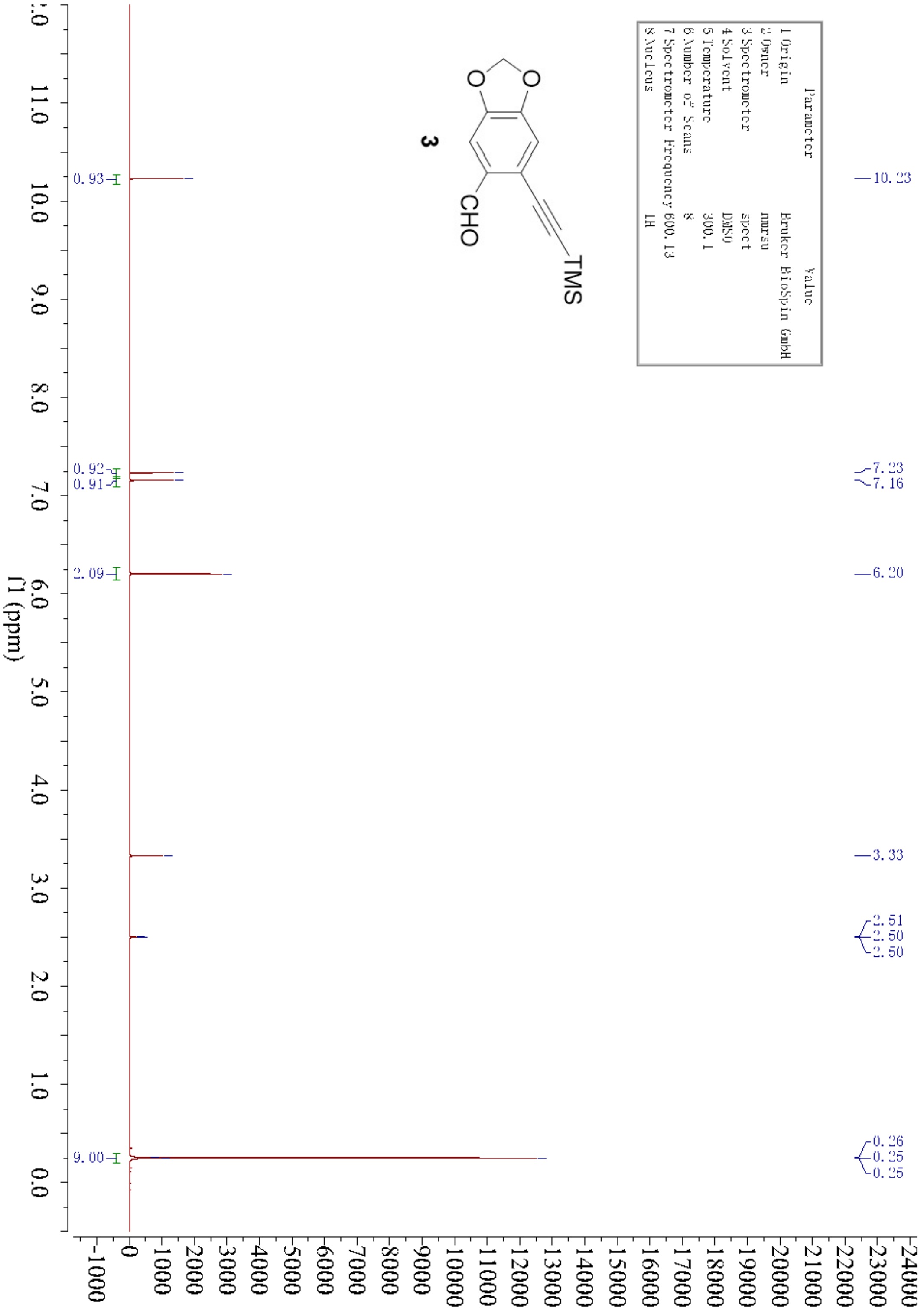

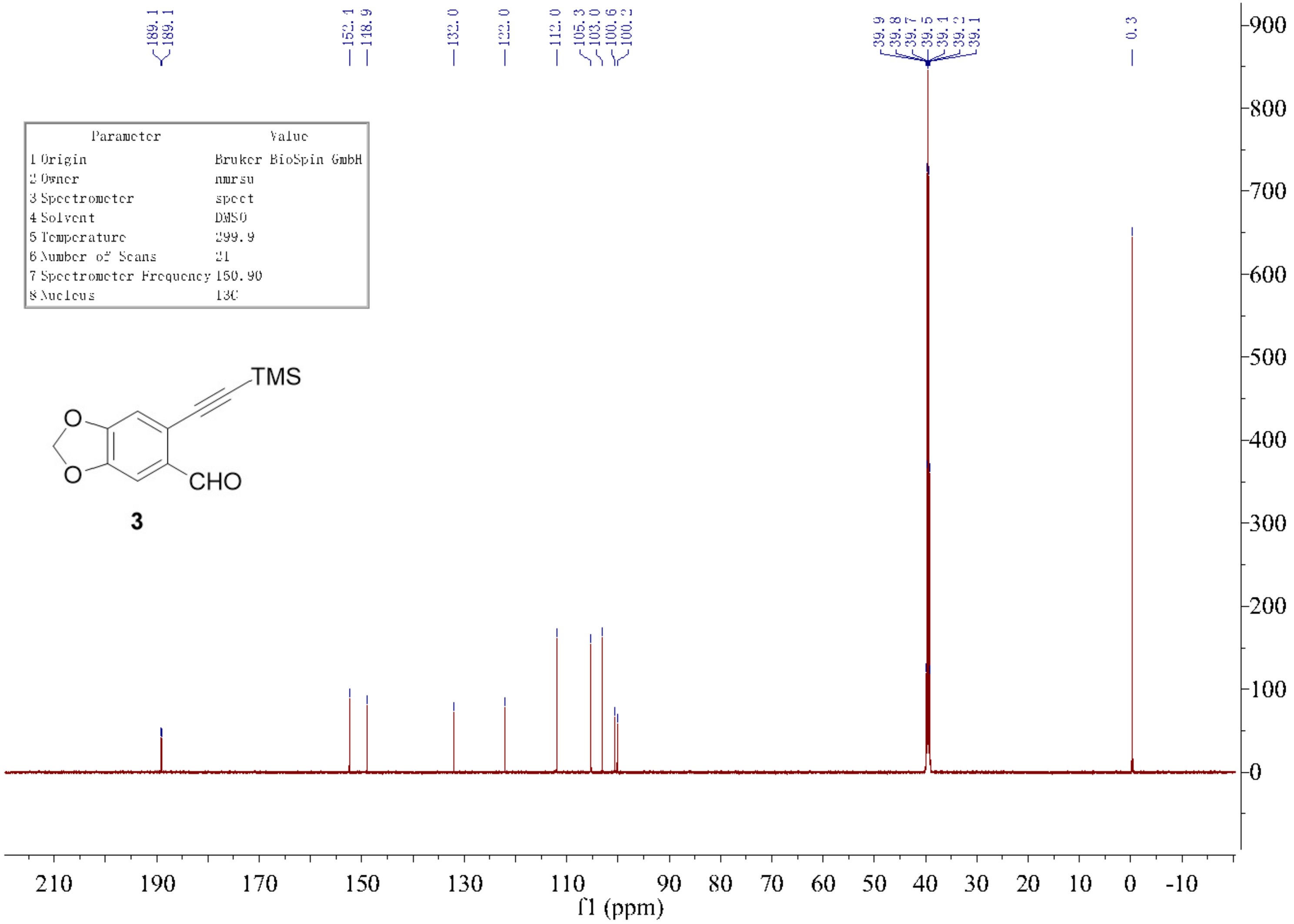
189.1
189.1
152.1
148.9
132.0
122.0
112.0
105.3
103.0
100.6
100.2
39.9
39.8
39.7
39.5
39.4
39.2
39.1
0.3
Parameter Value
1 Origin Bruker BioSpin GmbH
2 Owner nmrsu
3 Spectrometer spect
4 Solvent DMSO
5 Temperature 299.9
6 Number of Scans 21
7 Spectrometer Frequency 150.90
8 Nucleus 13C
TMS
CHO
O
O
3
f1 (ppm)

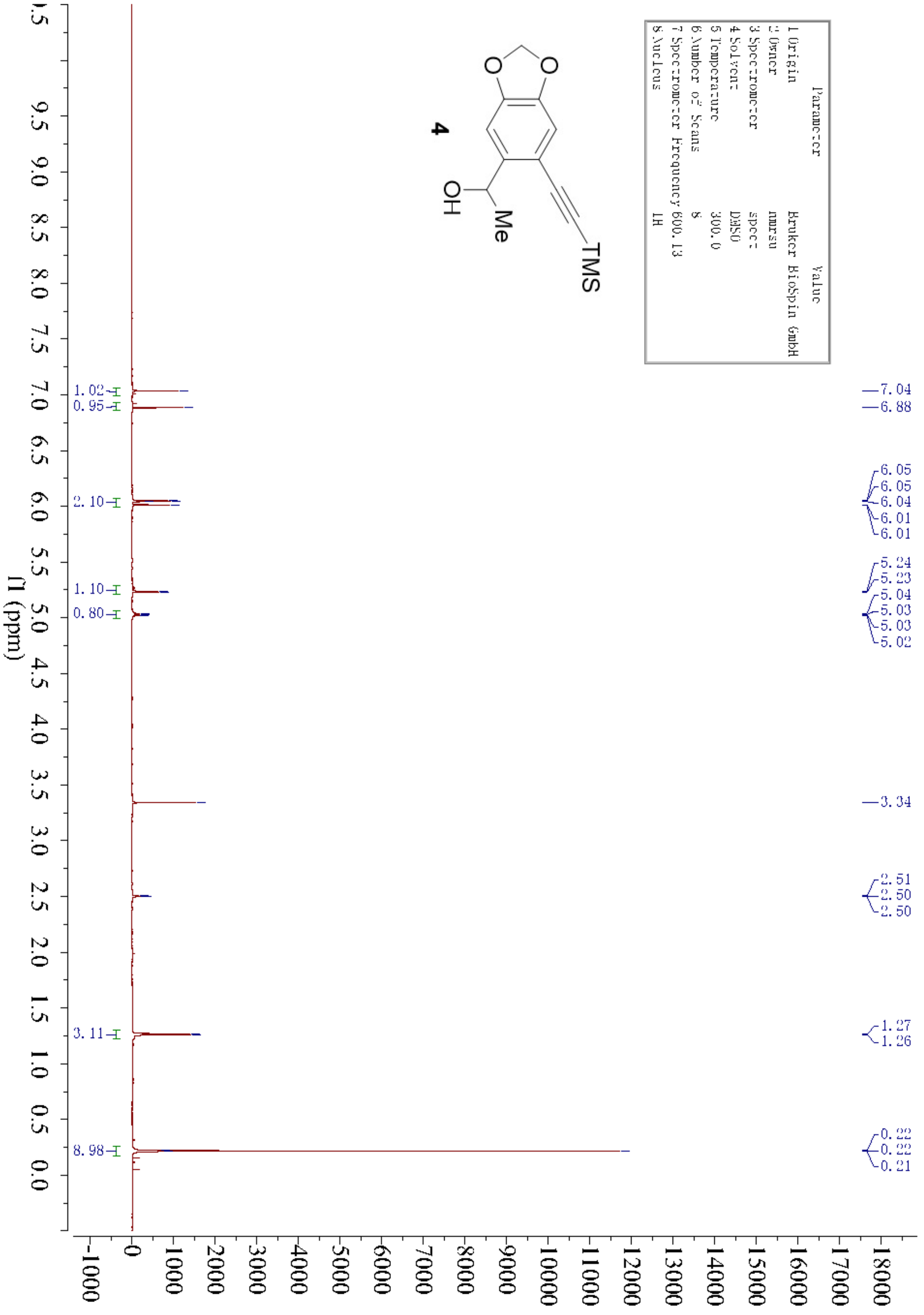
Parameter Value
1 Origin Bruker BioSpin GmbH
2 Owner nmrsu
3 Spectrometer spect
4 Solvent DMSO
5 Temperature 300.0
6 Number of Scans 8
7 Spectrometer Frequency 600.13
8 Nucleus 1H
TMS
Me
OH
O
O
4
7.04
6.88
6.05
6.05
6.04
6.01
6.01
6.01
5.24
5.23
5.04
5.03
5.03
5.02
3.34
2.51
2.50
2.50
1.27
1.26
0.22
0.22
0.21
1.02
0.95
2.10
1.10
0.80
3.11
8.98
f1 (ppm)

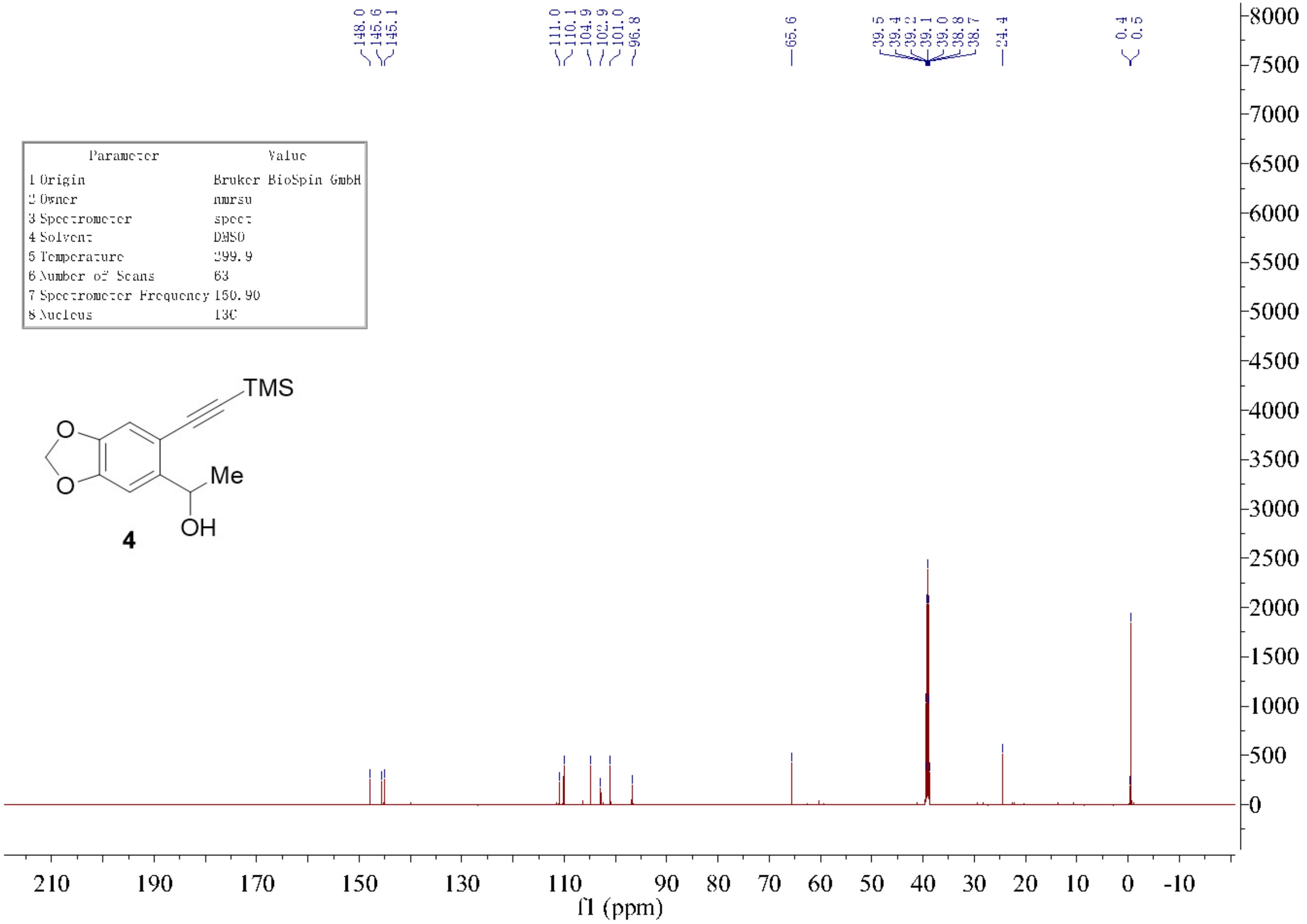
148.0
145.6
145.1
111.0
110.1
104.9
102.9
101.0
96.8
65.6
39.5
39.4
39.2
39.1
39.0
38.8
38.7
24.4
0.4
0.5
Parameter Value
1 Origin Bruker BioSpin GmbH
2 Owner nmrsu
3 Spectrometer spect
4 Solvent DMSO
5 Temperature 299.9
6 Number of Scans 63
7 Spectrometer Frequency 150.90
8 Nucleus 13C
TMS
O
O
Me
OH
4
f1 (ppm)

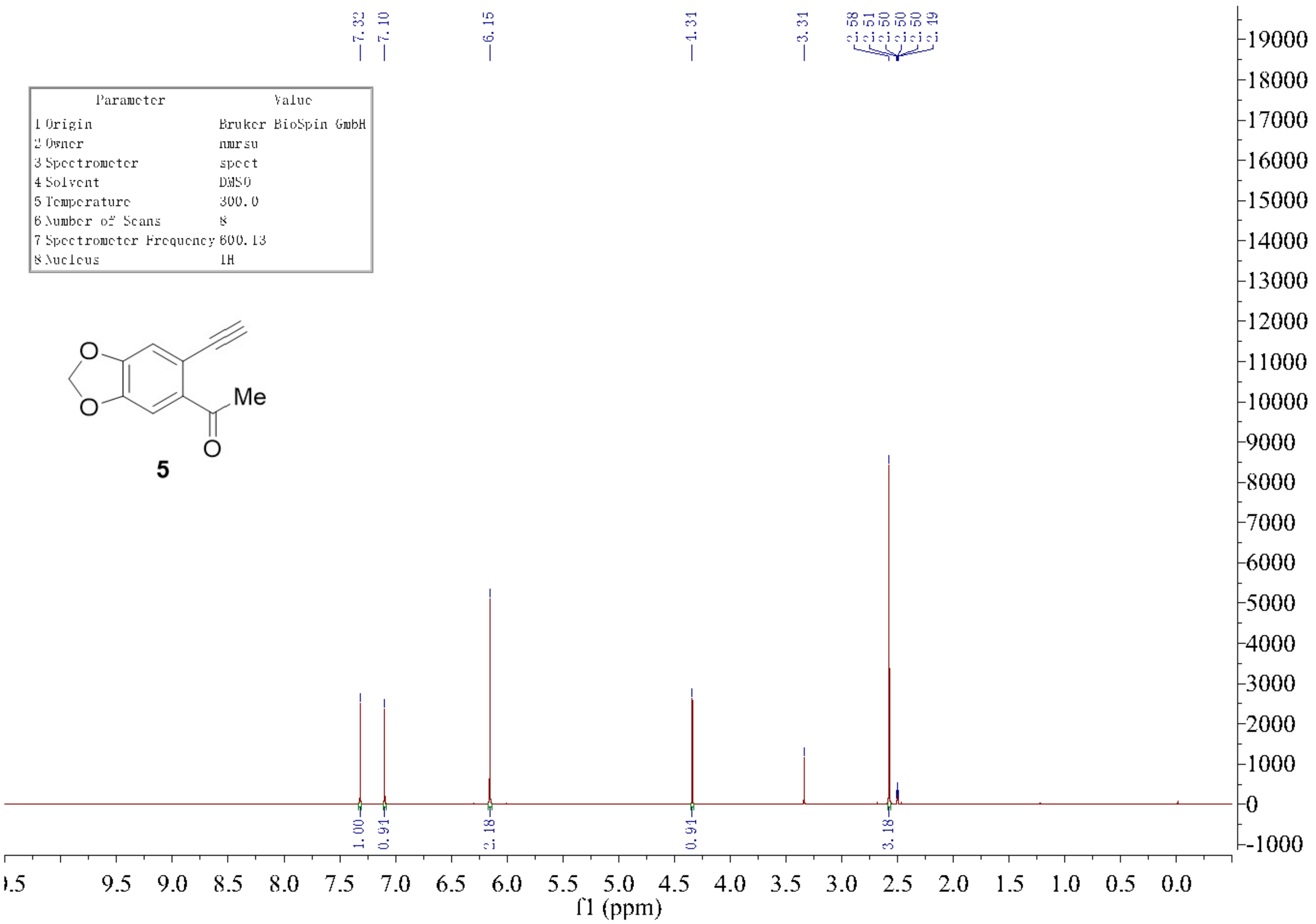
7.32
7.10
6.15
4.31
3.31
2.58
2.51
2.50
2.50
2.50
2.49
Parameter Value
1 Origin Bruker BioSpin GmbH
2 Owner nmrsu
3 Spectrometer spect
4 Solvent DMSO
5 Temperature 300.0
6 Number of Scans 8
7 Spectrometer Frequency 600.13
8 Nucleus 1H
Me
O
O
O
5
1.00
0.91
2.18
0.91
3.18
f1 (ppm)

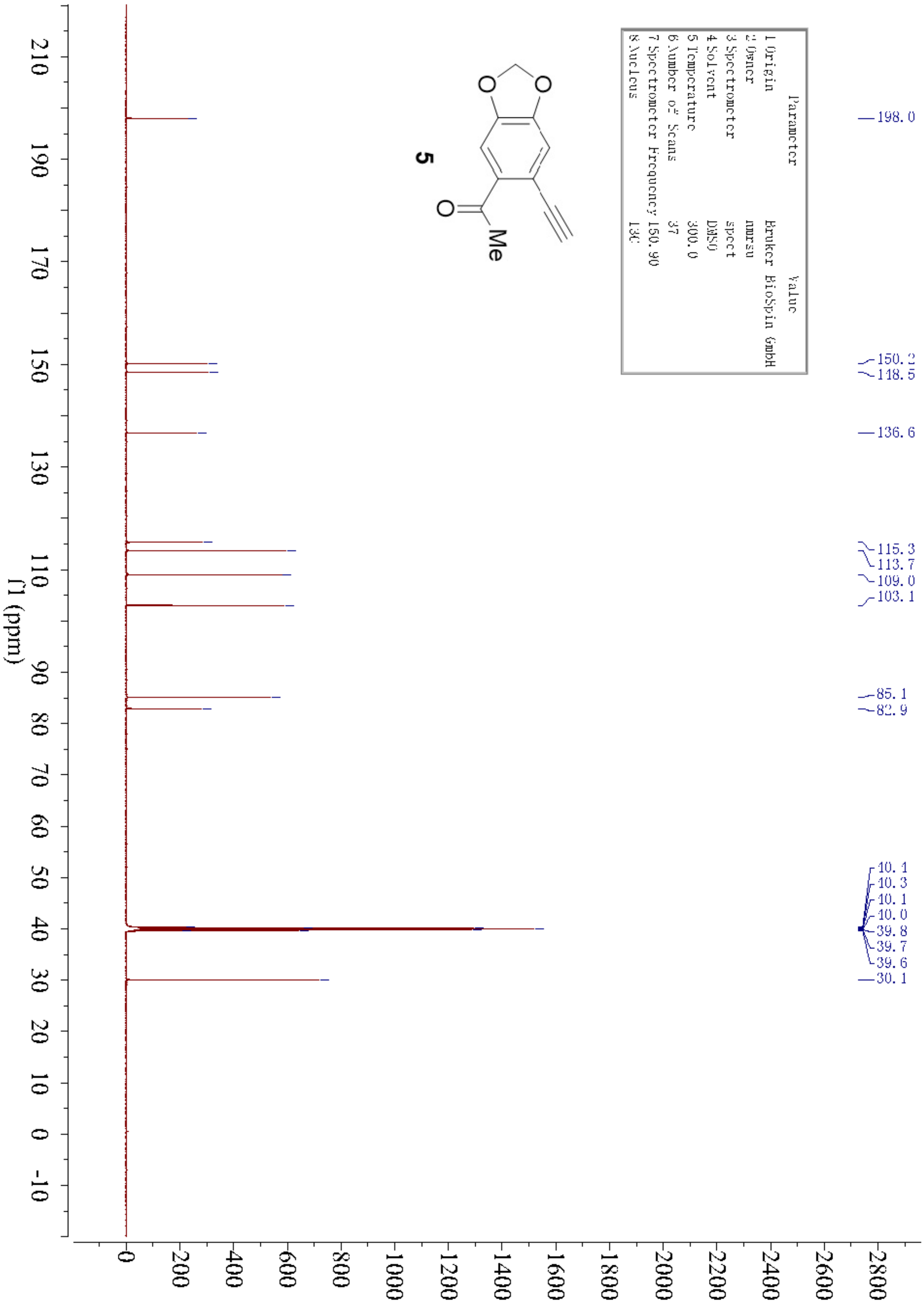

5
Me
O
Parameter
Value
1 Origin
Bruker BioSpin GmbH
2 Owner
nmrsu
3 Spectrometer
spect
4 Solvent
DMSO
5 Temperature
300.0
6 Number of Scans
37
7 Spectrometer Frequency
150.90
8 Nucleus
13C
198.0
150.2
148.5
136.6
115.3
113.7
109.0
103.1
85.1
82.9
40.1
40.3
40.1
40.0
39.8
39.7
39.6
30.1
f1 (ppm)

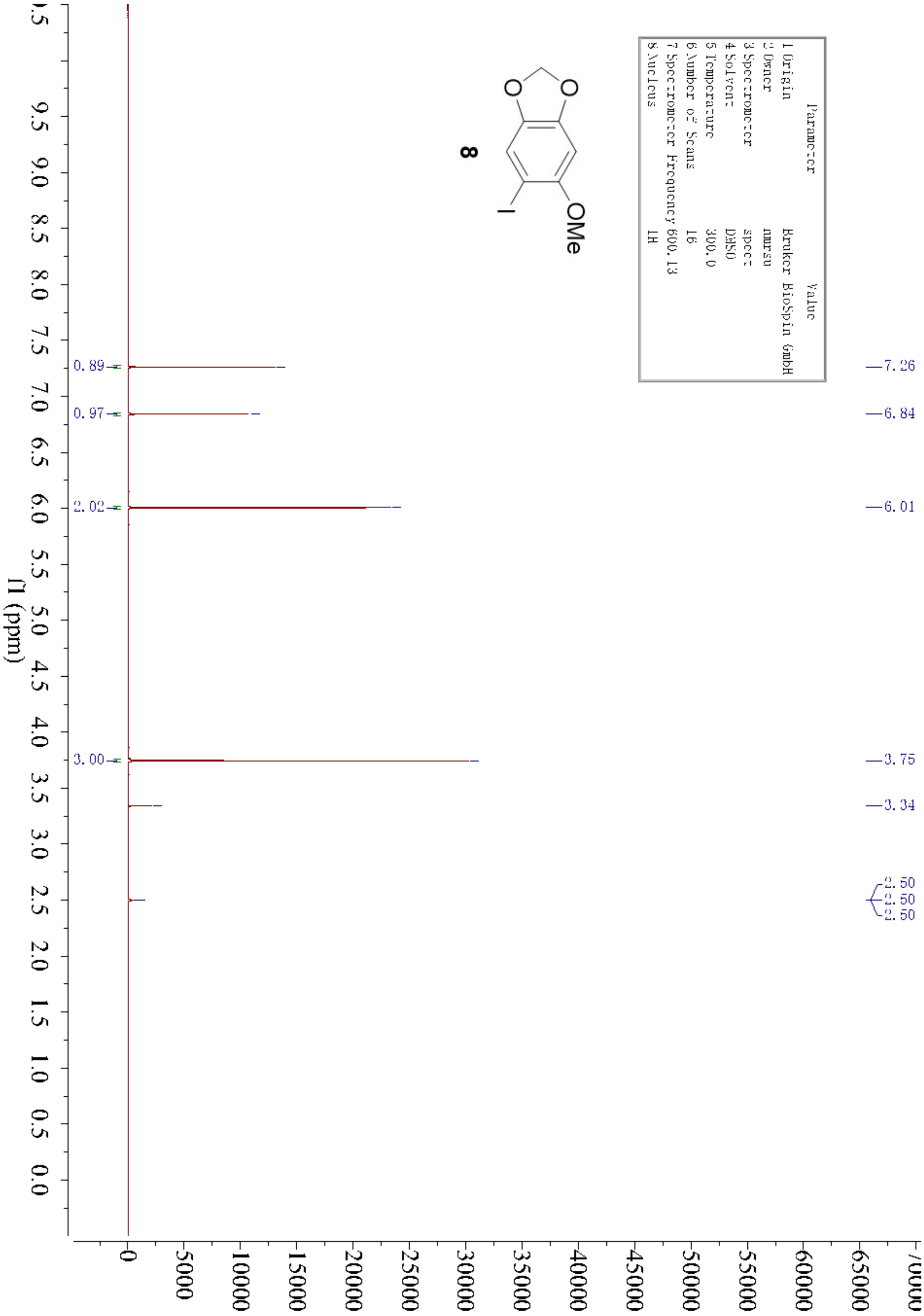
Parameter Value
1 Origin Bruker BioSpin GmbH
2 Owner nmrsu
3 Spectrometer spect
4 Solvent DMSO
5 Temperature 300.0
6 Number of Scans 16
7 Spectrometer Frequency 600.13
8 Nucleus 1H
OMe
I
O
O
8
7.26
6.84
6.01
3.75
3.34
2.50
2.50
2.50
0.89
0.97
2.02
3.00
f1 (ppm)

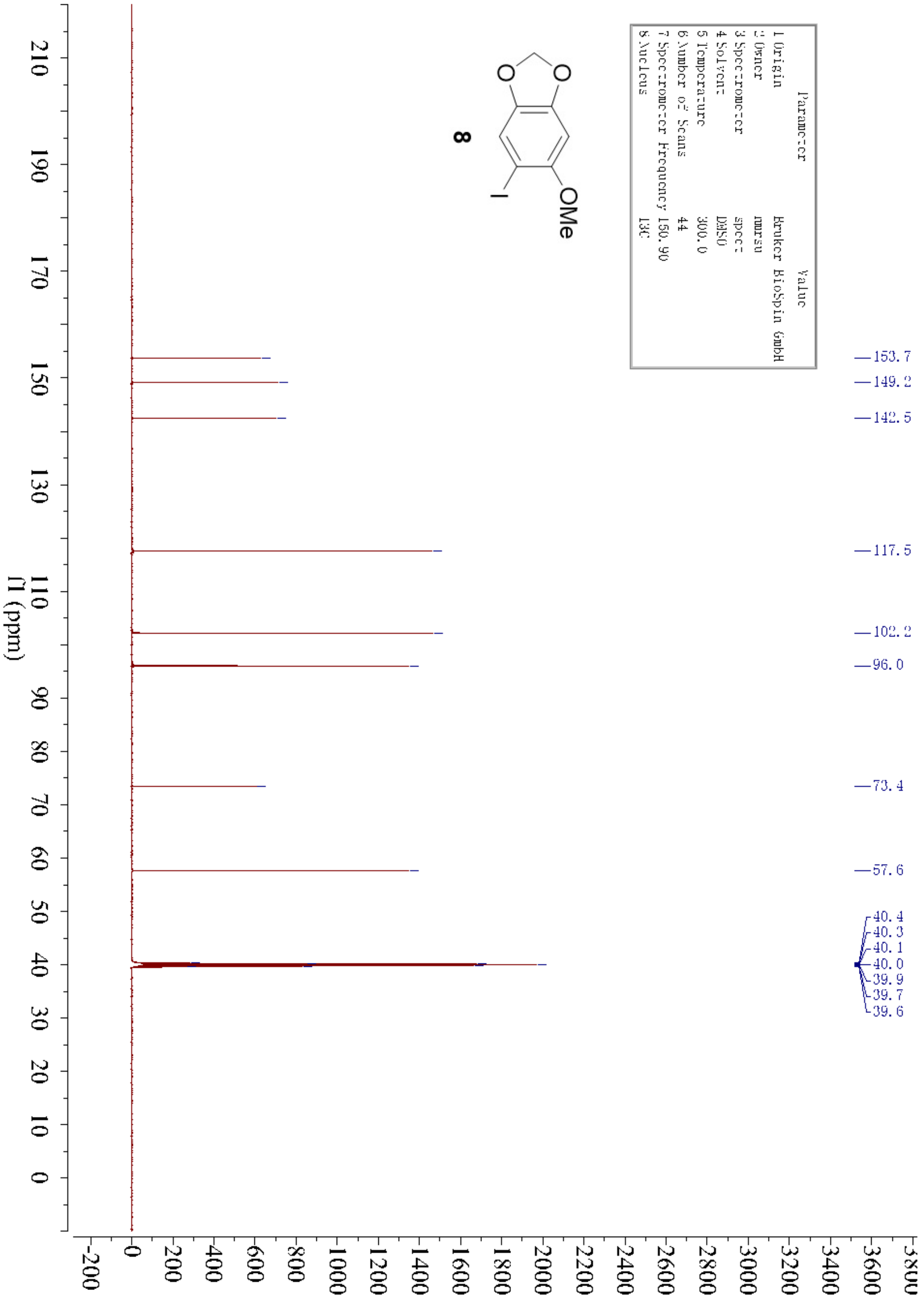
8
OMe
I
Parameter Value
1 Origin Bruker BioSpin GmbH
2 Owner nmrsu
3 Spectrometer spect
4 Solvent DMSO
5 Temperature 300.0
6 Number of Scans 44
7 Spectrometer Frequency 150.90
8 Nucleus 13C
153.7
149.2
142.5
117.5
102.2
96.0
73.4
57.6
40.4
40.3
40.1
40.0
39.9
39.7
39.6
f1 (ppm)

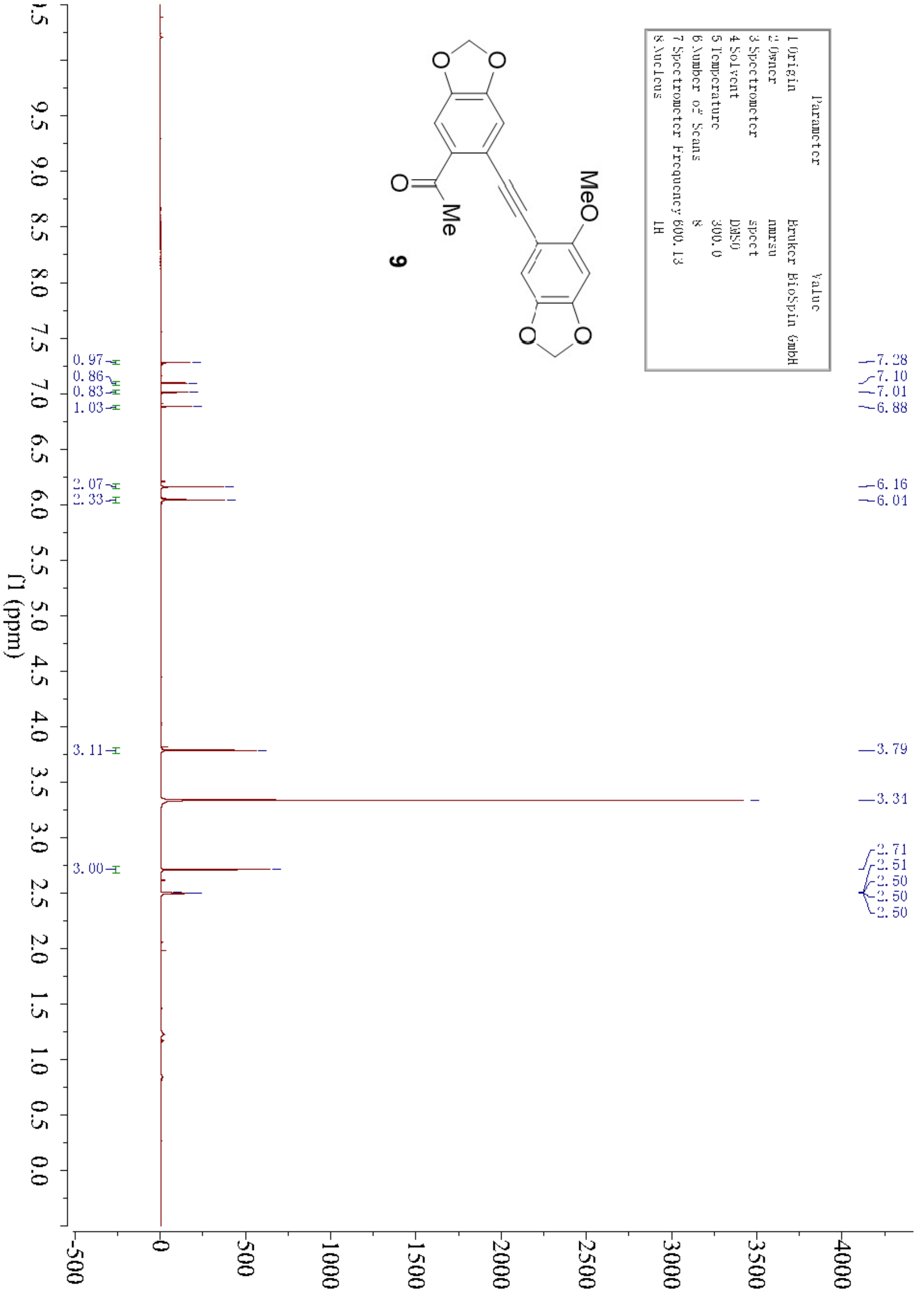
Parameter Value
1 Origin Bruker BioSpin GmbH
2 Owner nmrsu
3 Spectrometer spect
4 Solvent DMSO
5 Temperature 300.0
6 Number of Scans 8
7 Spectrometer Frequency 600.13
8 Nucleus 1H
MeO
Me
9
7.28
7.10
7.01
6.88
6.16
6.01
3.79
3.31
2.71
2.51
2.50
2.50
2.50
0.97
0.86
0.83
1.03
2.07
2.33
3.11
3.00
f1 (ppm)

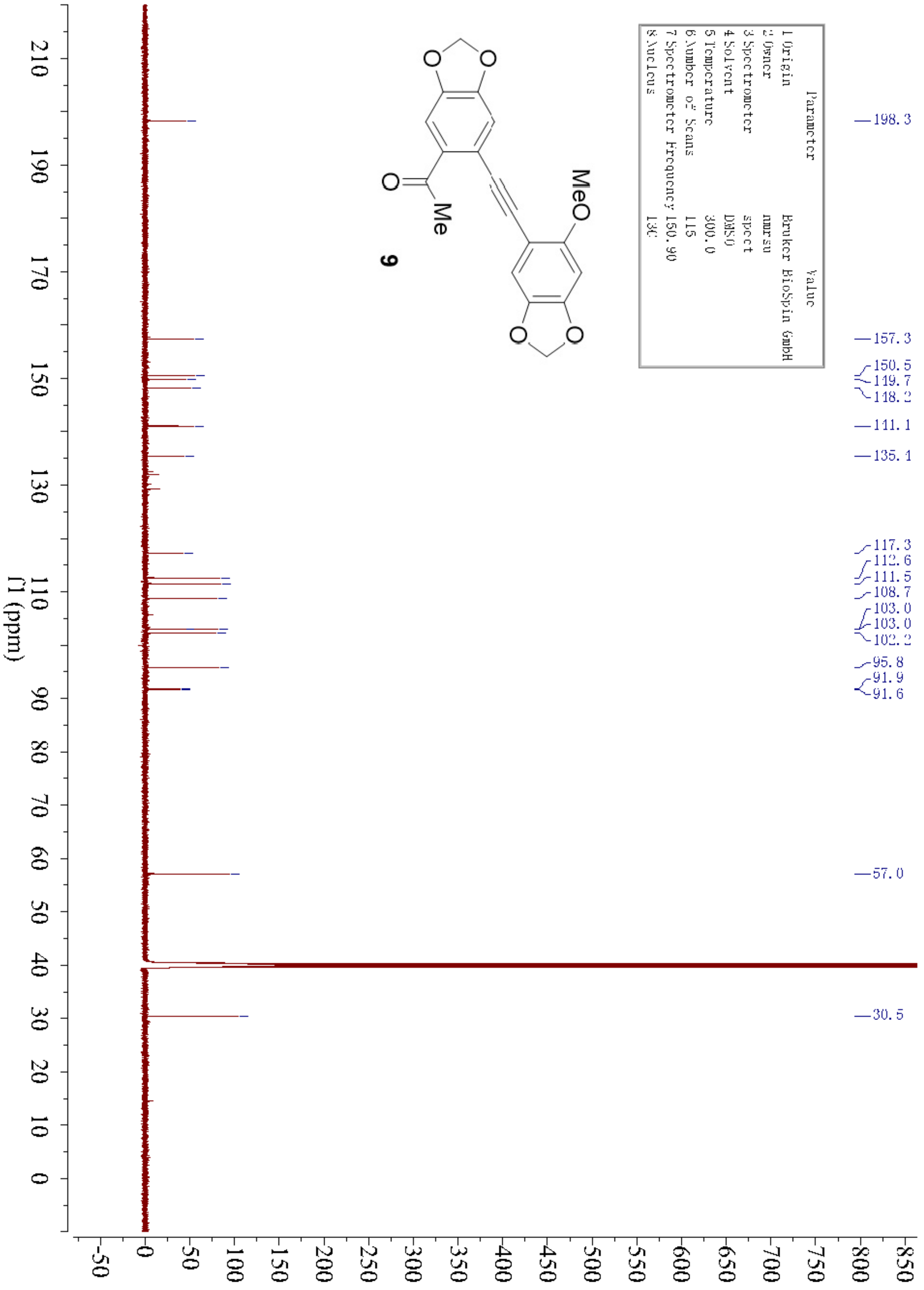
Parameter Value
1 Origin Bruker BioSpin GmbH
2 Owner nmrsu
3 Spectrometer spect
4 Solvent DMSO
5 Temperature 300.0
6 Number of Scans 115
7 Spectrometer Frequency 150.90
8 Nucleus 13C
MeO
O
O
O
O
O
Me
9
198.3
157.3
150.5
119.7
118.2
111.1
135.1
117.3
112.6
111.5
108.7
103.0
103.0
102.2
95.8
91.9
91.6
57.0
30.5
f1 (ppm)

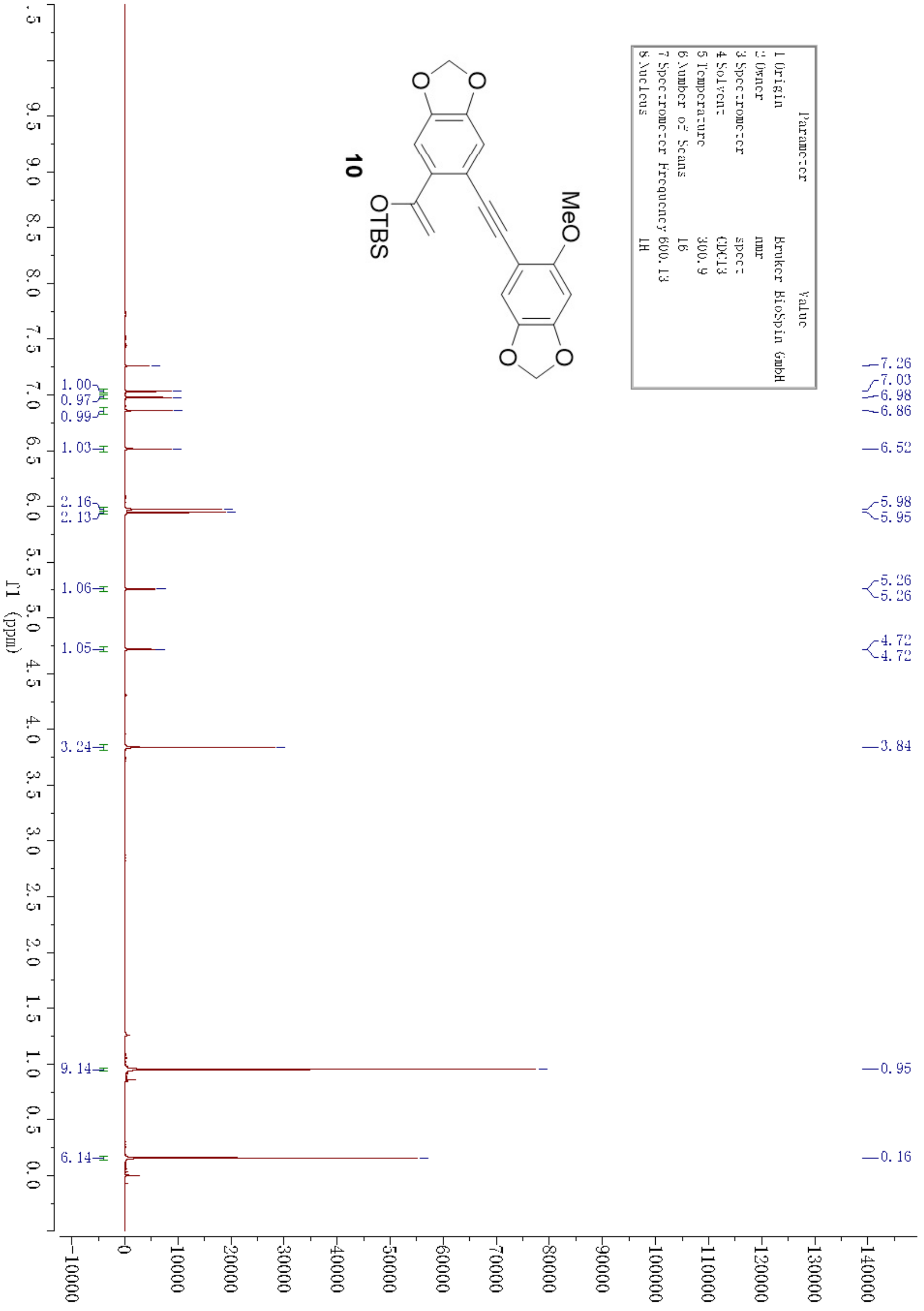
Parameter Value
1 Origin Bruker BioSpin GmbH
2 Owner nmr
3 Spectrometer spect
4 Solvent CDCl3
5 Temperature 300.9
6 Number of Scans 16
7 Spectrometer Frequency 600.13
8 Nucleus 1H
MeO
OTBS
10
7.26
7.03
6.98
6.86
6.52
5.98
5.95
5.26
5.26
4.72
4.72
3.84
0.95
0.16
1.00
0.97
0.99
1.03
2.16
2.13
1.06
1.05
3.24
9.14
6.14
f1 (ppm)

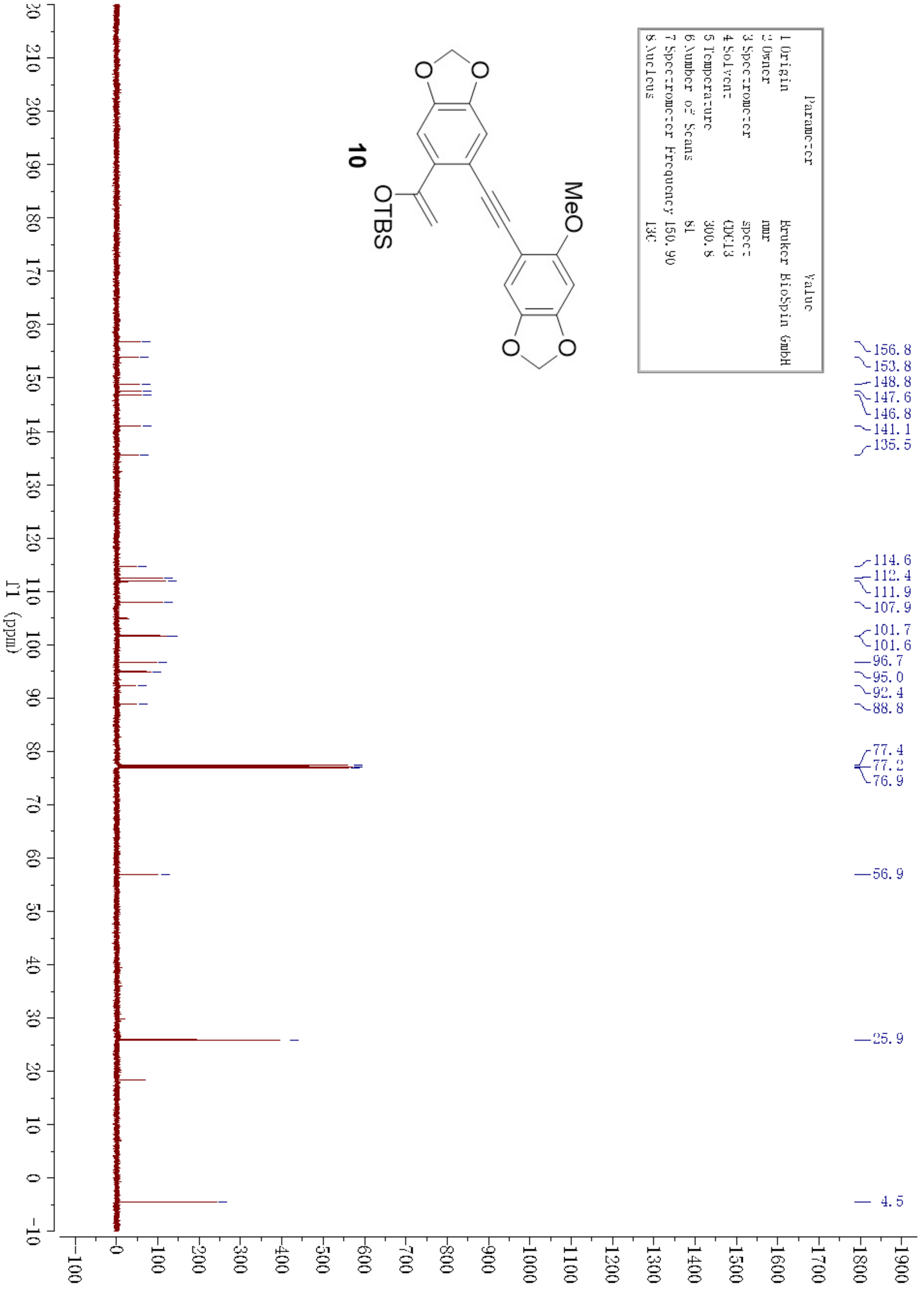

Parameter | Value
1 Origin | Bruker BioSpin GmbH
2 Owner | nmr
3 Spectrometer | spect
4 Solvent | CDCl3
5 Temperature | 300.8
6 Number of Scans | 81
7 Spectrometer Frequency | 150.90
8 Nucleus | 13C
MeO
OTBS
10
156.8
153.8
148.8
147.6
146.8
141.1
135.5
114.6
112.4
111.9
107.9
101.7
101.6
96.7
95.0
92.4
88.8
77.4
77.2
76.9
56.9
25.9
-4.5
f1 (ppm)

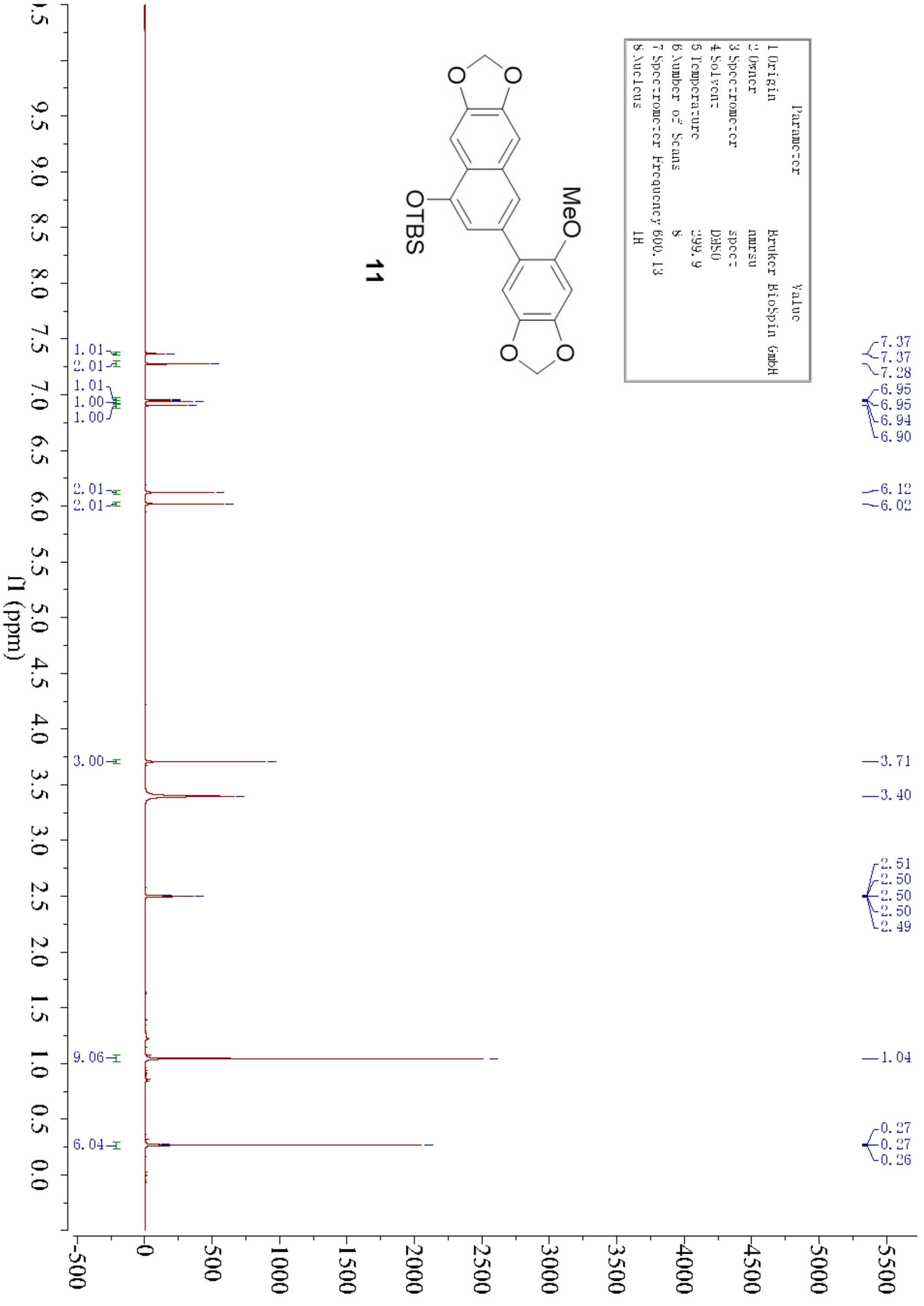

MeO
OTBS
11
f1 (ppm)

Parameter	Value
1 Origin	Bruker BioSpin GmbH
2 Owner	nmrsu
3 Spectrometer	spect
4 Solvent	DMSO
5 Temperature	300.0
6 Number of Scans	124
7 Spectrometer Frequency	150.90
8 Nucleus	13C

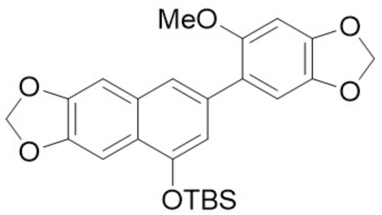

11

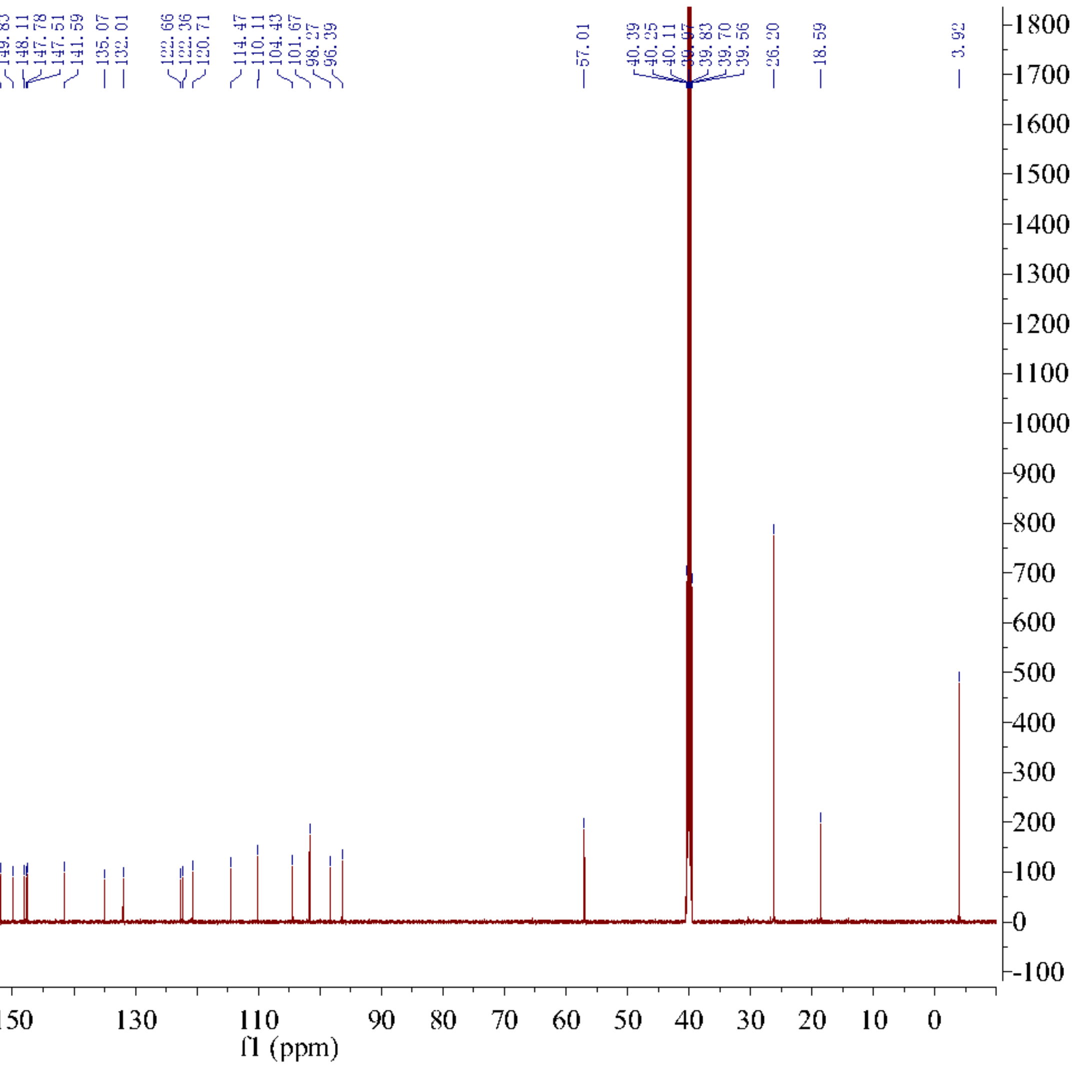

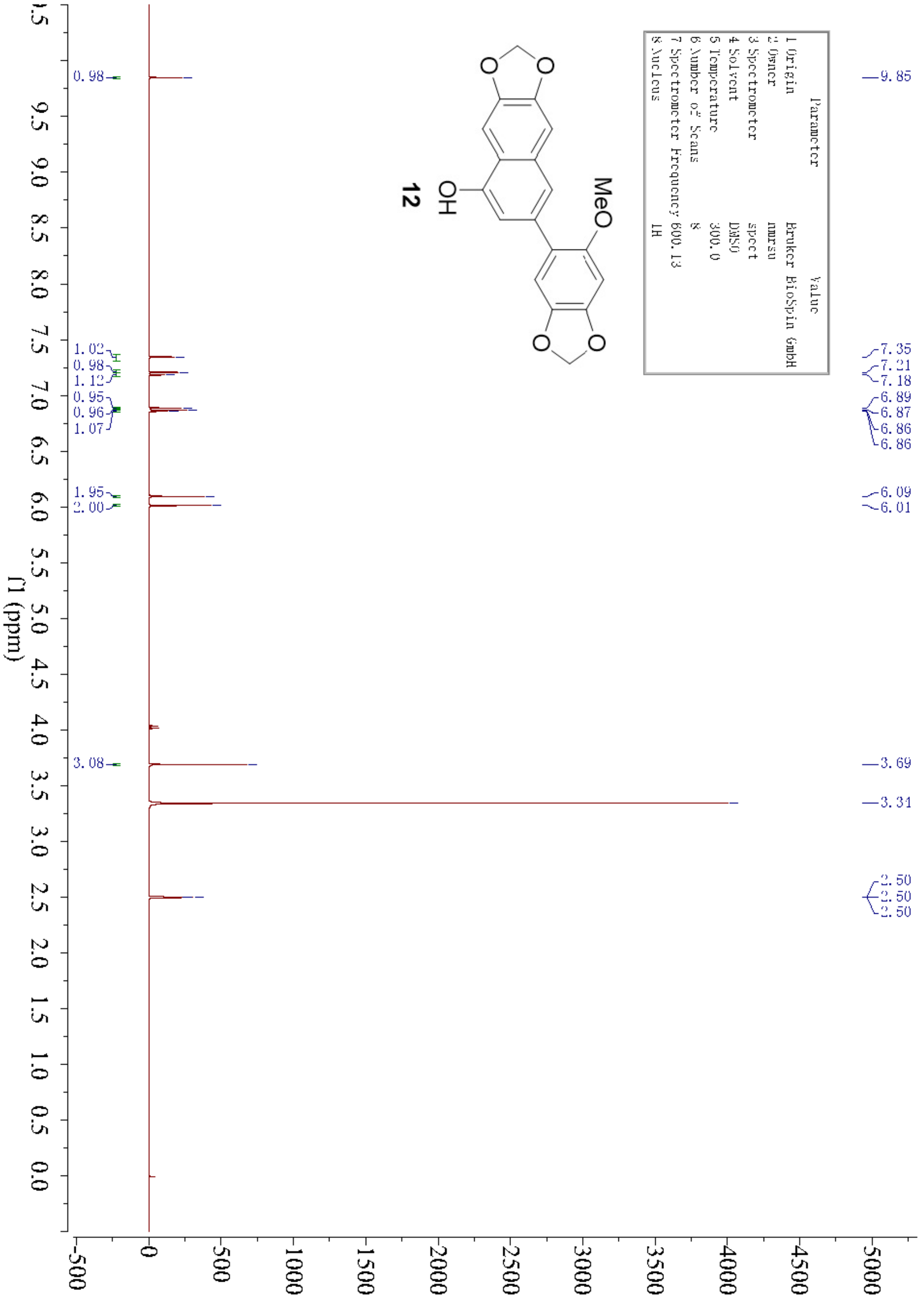
Parameter Value
1 Origin Bruker BioSpin GmbH
2 Owner nmrsu
3 Spectrometer spect
4 Solvent DMSO
5 Temperature 300.0
6 Number of Scans 8
7 Spectrometer Frequency 600.13
8 Nucleus 1H
MeO
OH
12
9.85
7.35
7.21
7.18
6.89
6.87
6.86
6.86
6.09
6.01
3.69
3.31
2.50
2.50
2.50
0.98
1.02
0.98
1.12
0.95
0.96
1.07
1.95
2.00
3.08
f1 (ppm)

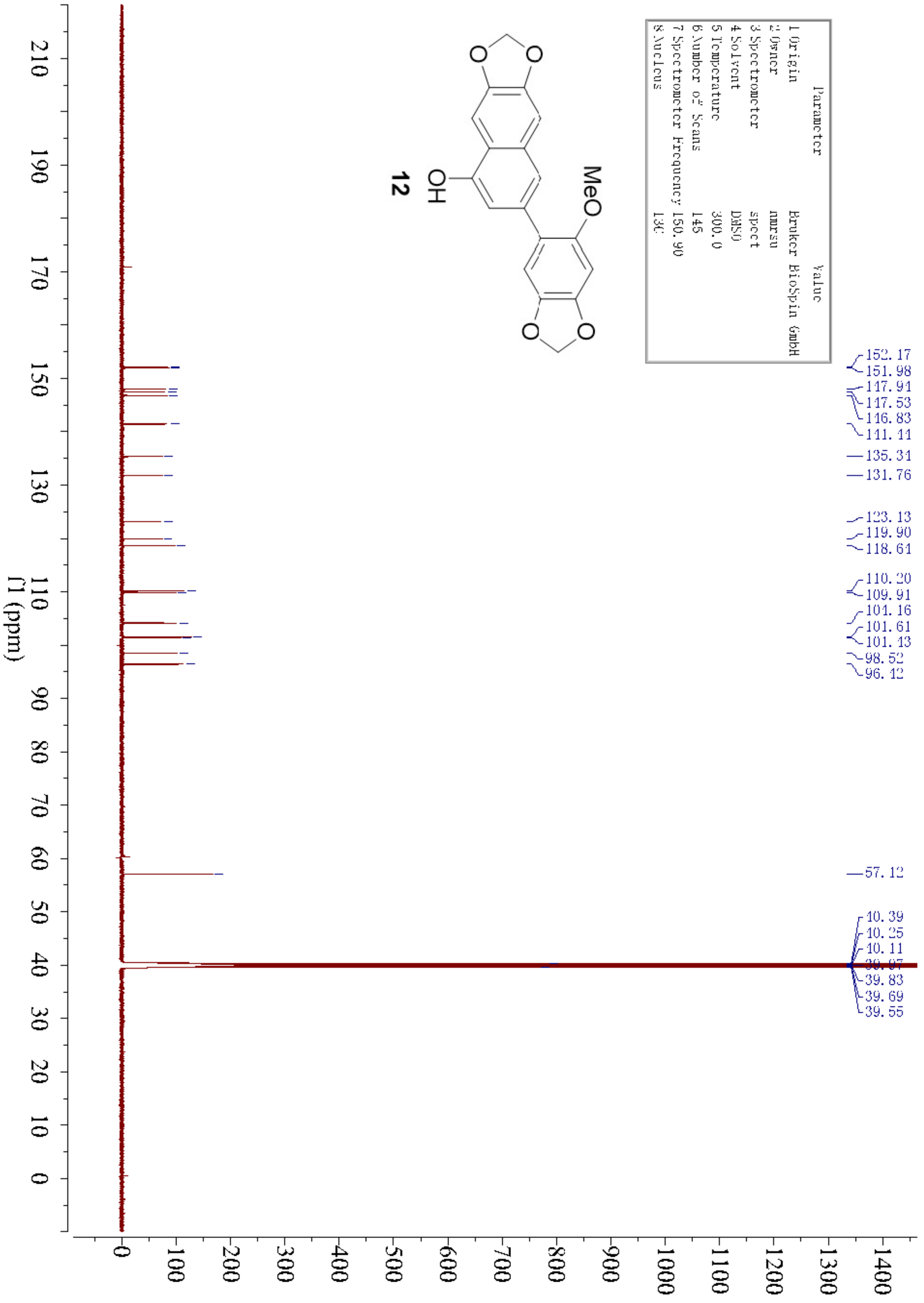

Parameter Value
1 Origin Bruker BioSpin GmbH
2 Owner nmrsu
3 Spectrometer spect
4 Solvent DMSO
5 Temperature 300.0
6 Number of Scans 145
7 Spectrometer Frequency 150.90
8 Nucleus 13C
MeO
OH
12
152.17
151.98
147.91
147.53
146.83
141.11
135.31
131.76
123.13
119.90
118.61
110.20
109.91
104.16
101.61
101.13
98.52
96.42
57.12
40.39
40.25
40.11
39.97
39.83
39.69
39.55
f1 (ppm)
